# RAB5A and TRAPPC6B are novel targets for Shiga toxin 2a inactivation in kidney epithelial cells

**DOI:** 10.1038/s41598-020-59694-w

**Published:** 2020-03-18

**Authors:** Ivan U. Kouzel, Alexander Kehl, Petya Berger, Ivan Liashkovich, Daniel Steil, Wojciech Makalowski, Yutaka Suzuki, Gottfried Pohlentz, Helge Karch, Alexander Mellmann, Johannes Müthing

**Affiliations:** 1 Institute for Hygiene and National Consulting Laboratory for Hemolytic Uremic Syndrome (HUS), University of Münster, D-48149 Münster, Germany; 2 Institute for Physiology II, University of Münster, D-48149 Münster, Germany; 3 Institute of Bioinformatics, University of Münster, D-48149 Münster, Germany; 4 Department of Computational Biology and Medical Sciences, University of Tokyo, 277-8562 Tokyo, Japan; 5 Present Address: Sars International Centre for Marine Molecular Biology, University of Bergen, 5008 Bergen, Norway

**Keywords:** Pathogens, RNAi, Transcriptomics

## Abstract

The cardinal virulence factor of human-pathogenic enterohaemorrhagic *Escherichia coli* (EHEC) is Shiga toxin (Stx), which causes severe extraintestinal complications including kidney failure by damaging renal endothelial cells. In EHEC pathogenesis, the disturbance of the kidney epithelium by Stx becomes increasingly recognised, but how this exactly occurs is unknown. To explore this molecularly, we investigated the Stx receptor content and transcriptomic profile of two human renal epithelial cell lines: highly Stx-sensitive ACHN cells and largely Stx-insensitive Caki-2 cells. Though both lines exhibited the Stx receptor globotriaosylceramide, RNAseq revealed strikingly different transcriptomic responses to an Stx challenge. Using RNAi to silence factors involved in ACHN cells’ Stx response, the greatest protection occurred when silencing RAB5A and TRAPPC6B, two host factors that we newly link to Stx trafficking. Silencing these factors alongside YKT6 fully prevented the cytotoxic Stx effect. Overall, our approach reveals novel subcellular targets for potential therapies against Stx-mediated kidney failure.

## Introduction

Gastrointestinal infections by enterohaemorrhagic *Escherichia coli* (EHEC), a highly pathogenic human subgroup of Shiga toxin (Stx)-producing *Escherichia coli*, cause haemorrhagic colitis and life-threatening extraintestinal complications such as haemolytic-uraemic syndrome (HUS)^1^. HUS is characterised by the triad of thrombocytopenia, microangiopathic haemolytic anaemia, and acute renal failure and can also lead to neurological sequelae^2,3^. Infections caused by EHEC are still a major global health threat, especially to children^4^, and antibiotic treatment is highly controversial^5,6^. Thus, no conclusive treatment is available beyond supportive care.

Mounting evidence has indicated that EHEC affects more cell types than originally thought. In the first step, EHEC attaches to the intestinal epithelium. There the cardinal virulence factor Stx — which occurs in two major types, Stx1 and Stx2, each of which have their own subtypes; subtype Stx2a is most often correlated with severe disease outcome^7^ — is translocated into the bloodstream by an incompletely understood mechanism^8^. After dissemination through the bloodstream, Stx enters the kidney and primarily targets microvascular endothelial cells of the glomeruli^9,10^, but it may also target renal epithelial cells^11,12^. The possible engagement of the renal epithelium in Stx intoxication was first indicated upon detecting the main Stx receptor, the glycosphingolipid (GSL) globotriaosylceramide (Gb3Cer; also known as CD77 [Galα1-4Galβ1-4Glcβ1-1Cer]), in the human kidney cortex and medulla^13^. Later, more evidence was found, specifically renal tubular injuries by Stx^14^ and Stx-induced apoptosis in kidney cortices of HUS patients and human renal tubular epithelial cells^15^. Subsequently, various renal epithelial cell types were found to be Stx-sensitive^16–19^. However, precisely how renal epithelial cells are affected by Stx is mostly unspecified.

Stx belongs to the family of AB_5_ toxins in which the A subunit is catalytically active and the B subunit binds to host targets^20–23^. Specifically, the Stx B subunit binds to Gb3Cer, the dominant receptor on human endothelial cells^12^, after which Stx is internalised by clathrin-dependent and -independent endocytic processes^22,24^ and then intracellularly trafficked in a variety of cells. In this process, Stx is transported in a retrograde manner, first from the early endosome (EE) to the *trans*-Golgi Network (TGN) and then via the Golgi apparatus to the endoplasmic reticulum (ER)^20–22,25,26^. During retrograde passage, the Stx A subunit is cleaved into the A1 and A2 fragments, after which the A1 fragment is retrotranslocated from the ER to the cytosol. Here, it exerts its ribosomal RNA *N*-glycosidase activity catalysing the depurination of a specific adenosine of the 28 S ribosomal RNA, which leads to the inhibition of protein biosynthesis and ultimately cell death^27,28^. Yet, while the uptake and trafficking process of Stx has been intensively studied, many features are still obscure and are under investigation^21,22,24^.

Interestingly, certain renal epithelial cell lines have been found to have different reactions to Stx: ACHN cells seem to have high sensitivity towards Stx^29^, but, in remarkable contrast, Caki-2 cells are nearly unresponsive to Stx^30^. As both cell lines similarly exhibit characteristics of renal distant tubular epithelial cells^31^, the reason for their opposing Stx sensitivities is confounding. One possibility is that these differences may be related to Stx receptor content and/or how Stx is taken up or trafficked in these cell lines.

To solve this apparent conundrum in Stx sensitivity and, thus, to generally elucidate the effect of Stx on renal epithelial cells, here we first reevaluated this contrasting phenotype. Next, we showed that the Stx receptor Gb3Cer is accessible and present in both ACHN and Caki-2 cells, indicating that differences in Stx sensitivity are not due to differences related to the Stx receptor. Then, to unravel the underlying cellular variation, we performed RNA sequencing (RNAseq). We found that upon Stx challenge, many genes exhibited profound differential expression, including trafficking-related genes. By applying RNA interference (RNAi), we were able to identify that knockdowns of RAB5A, TRAPPC6B, and YKT6 made highly sensitive ACHN cells practically refractory to Stx2a. Overall, this study shows that the distinct Stx sensitivity phenotypes of two similar renal epithelial cell lines serve as an excellent model for gaining insights into cellular susceptibility to Stx, and it proposes new targets for inactivating the cytotoxic action of Stx2a, potentially offering an avenue for future treatment of EHEC infections.

## Results

### ACHN cells are highly sensitive to Stx2a, while Caki-2 cells are largely refractory

**Figure 1:**
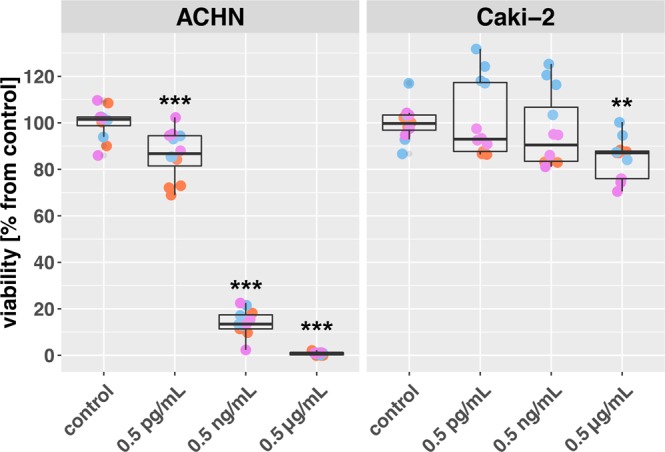
Differing susceptibility of ACHN and Caki-2 cells towards Stx2a. ACHN and Caki-2 cells were incubated for 72 h with increasing Stx2a concentrations as indicated. Cell viability represents percentage values compared to respective controls and is presented as boxplots: The central rectangle corresponds to the interquartile range, the horizontal line shows the median, and the “whiskers” are 1.5x of the interquartile range. Triplicate measurements of three biological replicates (*n* = 3) are shown in distinct colours (orange, magenta, and blue). Asterisks above boxplots denote the levels of significance in relation to the corresponding control: **p < 0.01; ***p < 0.001.

ACHN and Caki-2 cells were incubated with affinity-purified, endotoxin-free Stx2a (Fig. S1), the Stx subtype with the highest clinical relevance, in contrast to previous studies performed with Stx1^29,30^. Increasing Stx2a concentrations of 0.5 pg/mL, 0.5 ng/mL, and 0.5 μg/mL were used, and cell viability was determined after 72 h of toxin exposure, as shown in Fig. 1. Strikingly, ACHN cells were highly sensitive to Stx2a, showing a remarkable decrease in viability at 0.5 ng/mL Stx2a, where only 13.4% of cells survived (always compared to control cells); this was a drastic reduction from the cell viability of 86.8% at the particularly low Stx2a concentration of 0.5 pg/mL. At the highest Stx2a concentration of 0.5 μg/mL, only 1.0% of ACHN cells survived, indicating the cell culture had entirely collapsed. In contrast, Caki-2 cells were largely refractory to Stx2a exposure at 0.5 pg/mL and 0.5 ng/mL, showing only a negligible decrease in viability. Only at the highest concentration of 0.5 μg/mL was there a moderate but significant decrease to 87.1% survival. As ACHN cells only showed a comparable level of survival when exposed to the lowest concentration of 0.5 pg/mL Stx2a (86.8%), these results show that Caki-2 cells were 10^6^ times less susceptible to the toxin. Thus, ACHN and Caki-2 cells exhibit diametrically opposite Stx susceptibility.

### ACHN and Caki-2 epithelial cells exhibit virtually identical Gb3Cer profiles

To elucidate the observed sharp difference in toxin sensitivity of the two epithelial cell lines, we first examined the cellular binding of Stx2a and the presence of the main human Stx receptor, GSL Gb3Cer, which is required for binding and subsequent internalisation of the toxin^9^.

Applying fluorescence microscopy, we morphologically evaluated cell surface-bound Stx2a, using wheat germ agglutinin (WGA) as a common lectin for normalised prestaining of readily available carbohydrates as a surrogate for the total cell surface (Fig. S2). For ACHN cells, this showed either a rather weak but markedly punctate Stx2a staining (Fig. S2a,c), or a distinct toxin signal enrichment on the overall cell surface (Fig. S2a,d). For Caki-2 cells, a very uniform Stx2a staining was observed (Fig. S2b,e). Quantitative analysis of the toxin binding (for details see Methods) revealed that both cell lines exhibited a significant increase of the Stx2a/WGA fluorescence intensity ratio compared to the corresponding mock-stained samples: for Caki-2 cells the Stx2a-associated increase amounted to 0.13 above the background, whereas ACHN cells displayed a more robust increase of 0.58.

However, although one might think this could explain the cell lines’ differences in Stx sensitivity, the observed differences in toxin binding and/or cell accessibility actually cannot fully explain the strikingly distinct sensitivities, as even one single Stx molecule is sufficient to induce cell death in bound cells^32^.

**Figure 2:**
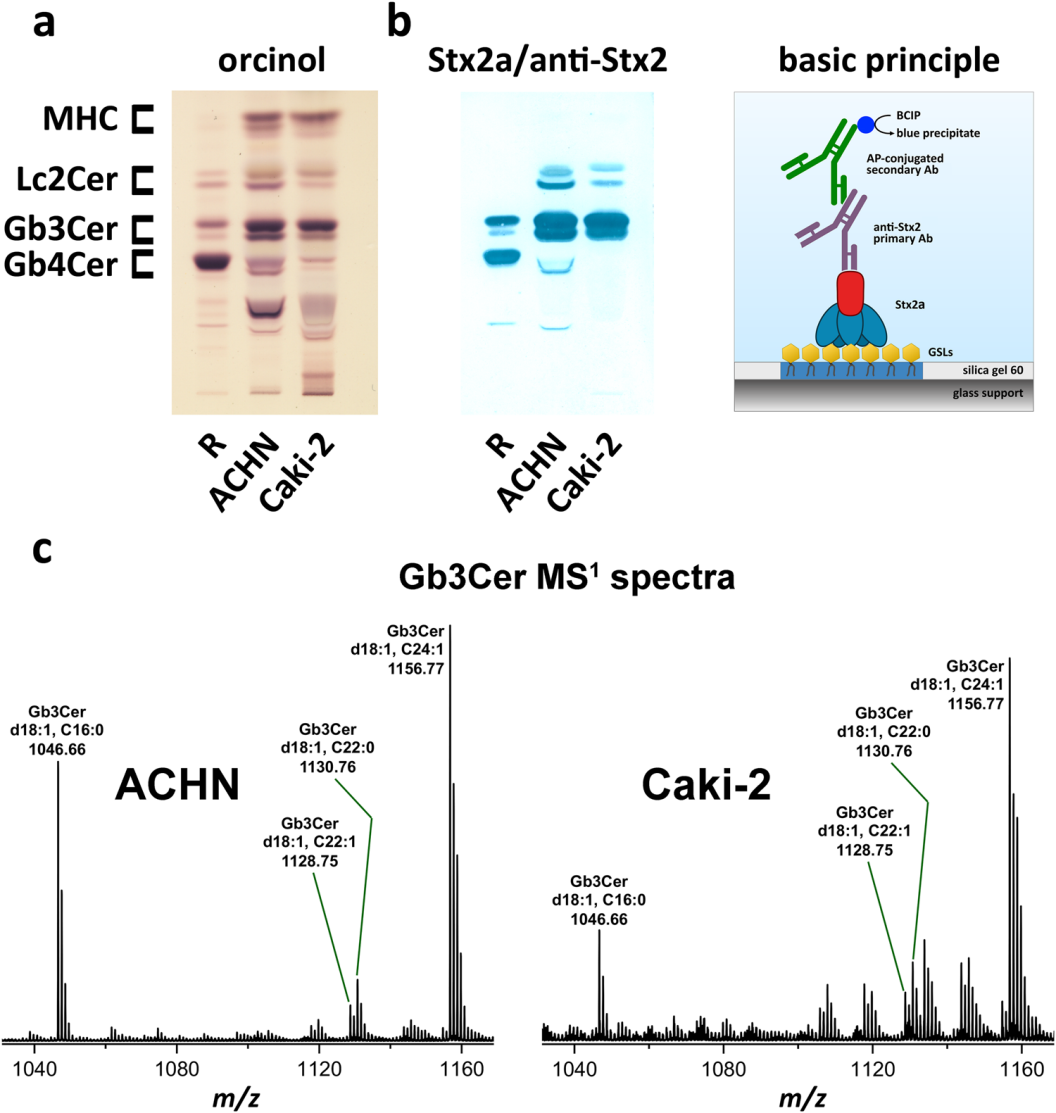
GSL profiles of ACHN and Caki-2 cells and mass spectra of Gb3Cer. (**a**) Orcinol stains of TLC-separated GSLs isolated from ACHN and Caki-2 cells. MHC – monohexosylceramide, Lc2Cer – lactosylceramide, Gb3Cer – globotriaosylceramide, Gb4Cer – globotetraosylceramide. GSLs applied in (**a**,**b**) correspond to 4 × 10^6^ cells and were separated alongside 10 µg (**a**) or 4 µg (**b**) of reference GSLs from human erythrocytes (R). (**b**) TLC overlay chromatogram and the scheme of the basic principle of Stx2a-mediated detection of GSL receptors. Bound anti-Stx2 antibodies were detected with alkaline phosphatase (AP)-conjugated secondary antibody (Ab) and 5-bromo-4-chloro-3-indolyl phosphate (BCIP) as substrate. (**c**) ESI mass spectra showing the various lipoforms of Gb3Cer of ACHN and Caki-2 cells.

To elucidate whether the differences in toxin binding are reflected in the global Stx receptor profiles, we next performed an in-depth investigation of the GSL composition of ACHN and Caki-2 cells. Initially, GSLs were extracted from ACHN and Caki-2 cells, and the overall GSL patterns were obtained by thin-layer chromatography (TLC) separation (Fig. 2a). The orcinol-stained GSLs suggested the presence of monohexosylceramide (MHC), lactosylceramide (Lc2Cer), Gb3Cer, and globotetraosylceramide (Gb4Cer), each with varying lipoforms separating as double bands on the chromatogram. Surprisingly, both cell lines showed comparable GSL patterns with minor differences in presumed Lc2Cer and Gb4Cer content as well as unknown GSLs separating below Gb4Cer (Fig. 2a). Because Stx2a preferentially binds to Gb3Cer^9^, we subsequently focused on Gb3Cer in solid phase binding assays and mass spectrometric characterisation.

Identical Gb3Cer double bands were immunochemically detected with Stx2a in TLC overlay assays for both cell lines (Fig. 2b). Afterwards, the exact structures of the various Gb3Cer lipoforms of ACHN and Caki-2 cells were identified using electrospray ionisation (ESI) mass spectrometry (MS) as shown with the MS^1^ spectra presented in Fig. 2c. Both ACHN and Caki-2 cell lines exhibit variable content of Gb3Cer species due to ceramide moieties harbouring d18:1 sphingosine linked to a C16:0, C22:0, C22:1, or C24:1 fatty acid. Verification of the proposed structures was done by collision-induced (CID) MS, and representative MS^2^ spectra of identified Gb3Cer species with fragmentation schemes are shown for ACHN and Caki-2 cells in Fig. S3a,b, respectively. However, we measured here total GSLs in the cellular extracts and it was not possible to distinguish between GSLs exposed on the cell surface and GSLs localized intracellularly.

Collectively, even though ACHN and Caki-2 cells show topographically different cellular Stx attachment, they display comparable Gb3Cer lipoforms, as shown by TLC Stx2a overlay immunoassays and verified by MS analysis. This indicates that additional factors are likely contributing towards the cell lines’ contrasting Stx sensitivity.

### RNAseq reveals profound gene expression differences between ACHN and Caki-2 cells in response to Stx2a

**Figure 3:**
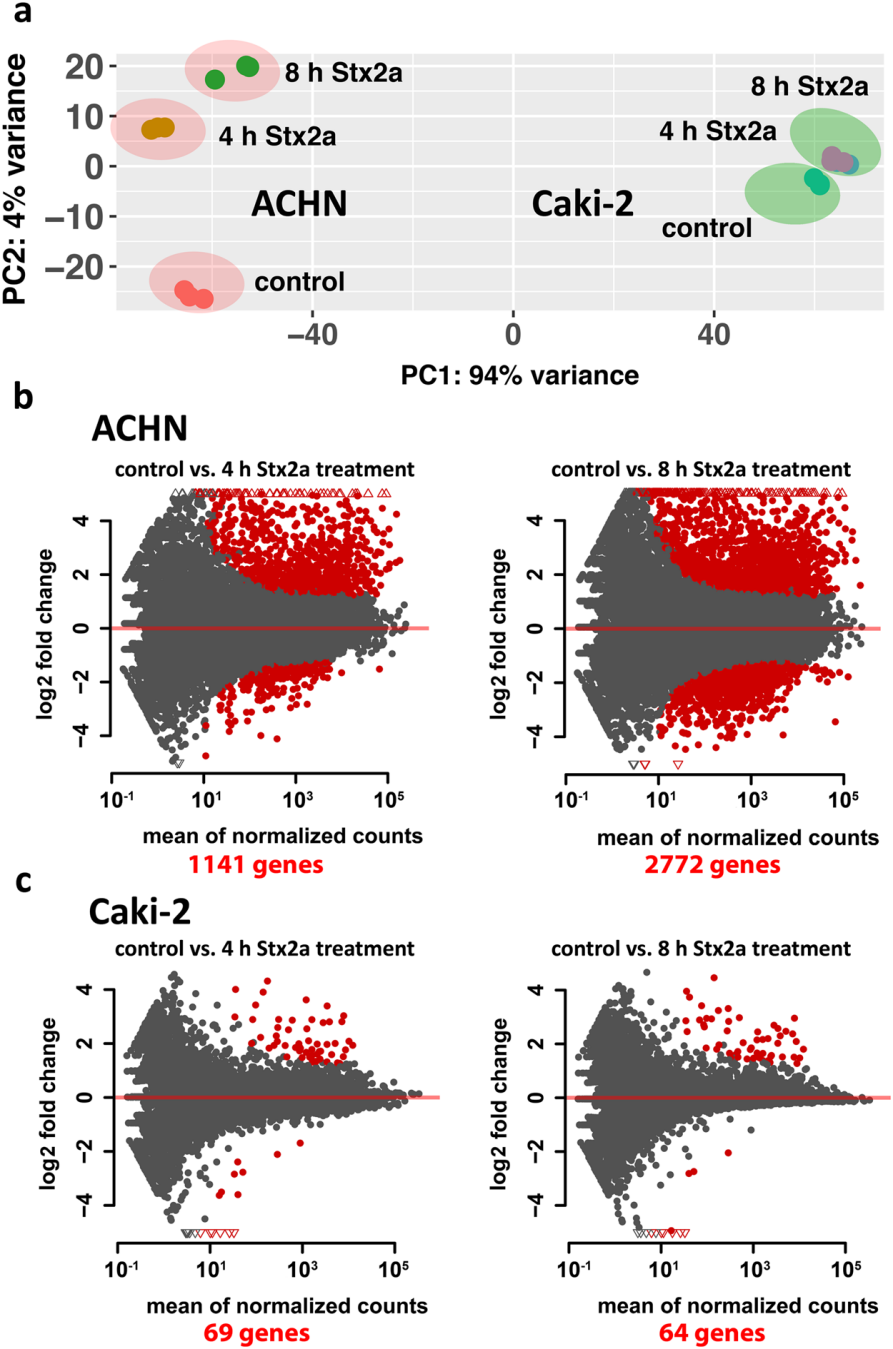
Differential gene expression of Stx2a-sensitive ACHN and Stx2a-refractory Caki-2 cells upon Stx2a exposure in comparison to untreated cells. (**a**) PCA of RNAseq data for ACHN and Caki-2 cells under different conditions, i.e. exposure to Stx2a for 4 h or 8 h and untreated control. The respective conditions with three biological replicates each are portrayed with distinct colours. (**b**,**c**) MA plots of genes in ACHN cells (**b**) or Caki-2 cells (**c**) two times or more up- or down-regulated after 4 h or 8 h of Stx2a challenge, respectively. Statistically significant genes out of ≈21,000 with nonzero total read count (*p* < 0.01) with an at least twofold change are plotted in red. Triangles indicate genes outside the fold change scale.

To gain cell physiological insights into the opposing susceptibility of the Stx-sensitive ACHN versus the *de facto* Stx-refractory Caki-2 cells, we performed RNAseq. The cells were exposed to Stx2a for 4 h or 8 h, and the results were compared to a control at starting conditions without Stx2a. An overall sequencing summary of the RNAseq run is presented in Table S1. Next, we performed exploratory data analysis and assessed the overall similarity between the two cell lines and between 4 h or 8 h Stx2a exposure and the control by applying principal component analysis (PCA). PCA revealed, first, a clear distinction between ACHN and Caki-2 cells globally. Second, it also revealed a distinction between Stx2a exposure and control conditions, though the difference was more pronounced with ACHN cells than with Caki-2 cells (Fig. 3a).

Then, we examined the transcriptomic response to Stx2a exposure in more detail, disregarding those genes with a less than twofold difference versus control conditions. Toxin-sensitive ACHN cells exhibited a generally strong response, with 1,141 significantly differentially expressed genes upon 4 h Stx2a exposure; many of these genes were highly upregulated, and the ratio of up- to downregulated genes was 4.1 (Fig. 3b, left panel). Prolonging Stx2a exposure to 8 h more than doubled this number, resulting in 2,772 significantly differentially expressed genes, and the up-to-down ratio was reduced to 1.8 (Fig. 3b, right panel). In contrast, toxin-refractory Caki-2 cells showed only a mild response, with 69 genes at 4 h Stx2a exposure of which many were highly downregulated; still, the up-to-down ratio was 4.0 (Fig. 3c, left panel). Exposing Caki-2 cells to Stx2a for 8 h altered this number only marginally to 64 genes, and the up-to-down ratio increased to 5.5 (Fig. 3c, right panel).

**Figure 4:**
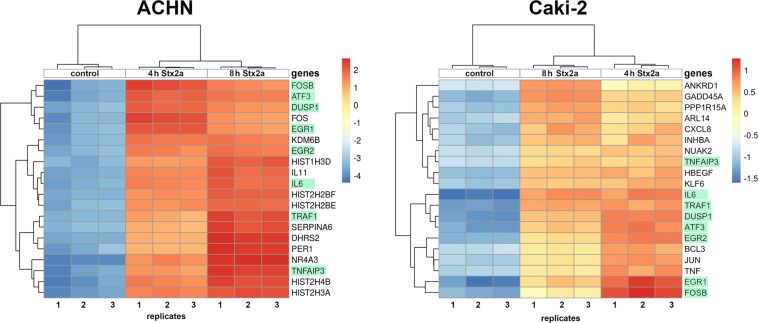
Heatmaps of the top 20 genes with the highest variability in ACHN and Caki-2 cells when exposed to Stx2a. Heatmap colour corresponds to the amount by which a gene expression variance deviates from the gene’s mean variance across all nine samples in each cell line with three samples per condition, i.e. 4 h or 8 h Stx2a exposure and untreated control. Genes and conditions are clustered according to their sample similarities. The 8 genes shared by both ACHN and Caki-2 cells in the top 20 list (out of ≈21,000 genes) are highlighted in green.

Interestingly, looking more closely at the top 20 genes with the highest variability in ACHN and Caki-2 cells across conditions (Fig. 4) shows that 8 out of these 20 genes upregulated by Stx2a exposure are shared by both cell lines, namely: FOSB, ATF3, DUSP1, EGR1, EGR2, IL6, TRAF1, and TNFAIP3. This highlights that both cell lines react to Stx2a exposure. Altogether, despite some overlap, a much stronger transcriptomic response to Stx2a of toxin-sensitive ACHN cells was detected when compared to toxin-resistant Caki-2 cells.

### Interaction analysis discloses clusters of differentially expressed genes of ACHN versus Caki-2 cells in retrograde trafficking

As our main objective was to decipher the cellular attributes responsible for these cell lines’ diverging sensitivity to Stx, we performed time series analysis that included in the statistical model both ACHN and Caki-2 cells as well as the three experimental conditions, i.e. Stx2a exposure for 4 h, for 8 h, or no Stx2a exposure (control). The output of this analysis was a list of genes which behaved in a cell-specific manner over time. Genes in both ACHN and Caki-2 cells that similarly moved up or down in response to Stx2a were not given small *p*-values by the model and, thus, were excluded from further analysis (for details see Methods). By analysing the top 2,000 differentially expressed genes of ACHN versus Caki-2 cells, it became apparent that many play a role in endocytosis and intracellular retrograde trafficking of Stx^21,22^. In general, the involved host proteins comprise numerous vesicle lifecycle factors, i.a. RAB GTPases such as RAB6A as the master regulator, or cytoskeleton components like the microtubules and its minus-end-directed motor protein complex dynein for transport. Furthermore, Stx trafficking also involves vesicle tethering factors such as golgins or the conserved oligomeric Golgi (COG) or Golgi-associated retrograde protein (GARP) complexes and different SNAREs, which are membrane fusion mediators. Genes from the top 2,000 list representing such host factors are summarised in Table 1 (for the corresponding references see Table S2). Consequently, our approach demonstrates a valid way to explore potential cellular differences in Stx sensitivity, and it prompted us to investigate trafficking-related genes in detail.

**Table 1:** Selected target genes of ACHN and Caki-2 cells and their biological role.

gene symbol	biological role	cluster in Figure 5^a^
*genes with higher expression in ACHN cells and a known role in Stx trafficking*		
ATP6V0A1, ATP6V0D1, and APT6V1G1	vacuolar ATPase components	─
BICD1	RAB6A- and dynein-interacting golgin/CCT^b^	─
BNIP1	ER-localised SNARE, in complex with STX18/USE1/SEC22B	i
COG3/SEC34	component of the Golgi-localised COG^c^ MTC^d^	i
DYNLL1	one of the light chains of the microtubule motor dynein	─
GOLGA1/golgin-97	ARL1-interacting golgin/CCT	i
GOSR1/GS28	Golgi-localised SNARE, in complex with STX5/BET1L/YKT6	i
STX5	Golgi-localised SNARE, in complex with GOSR1/BET1L/YKT6	i
TBC1D17	deactivating RAB8 and RAB21 GAP^e^	i
VAMP2	plasma membrane-localised SNARE	i
VPS11	core component of the endosomal CORVET^f^/HOPS^g^ MTCs, RAB5 effector	─
*genes with higher expression in Caki-2 cells and a known role in Stx trafficking*		
ARL1	Golgi-localised small GTPase	─
BICD2	RAB6A-interacting golgin/CCT	─
RAB11A	small GTPase, master regulator of the recycling endosome	─
*genes with higher expression in ACHN cells and an unknown role in Stx trafficking*		
DCTN4	component of the integral dynein-interacting dynactin complex	─
DENND5A/RAB6IP1	putative activating RAB6 GEF^h^	─
NEDD4	ubiquitin ligase, part of the ESCRT^i^ pathway	ii
NEDD4L	ubiquitin ligase, part of the ESCRT pathway	ii
RAB5A	small GTPase, master regulator of the early endosome	i
RAB9A	late endosomal small GTPase	i
RABGEF1/rabex-5	major activating RAB5 GEF	i
TRAPPC6B	core component of the Golgi-localised TRAPP^j^ MTCs	i
TSG101	ESCRT-I component	ii
USE1	ER-localised SNARE, in complex with STX18/BNIP1/SEC22B	i
*other genes analysed with a known role in Stx trafficking*		
BET1L/GS15	Golgi-localised SNARE, in complex with STX5/GOSR1/YKT6	—
NBAS/NAG	component of the ER-localised NRZ^k^ MTC	—
RAB6A	small GTPase, master regulator of *intra*-Golgi trafficking	—
VTI1A	endosomal/Golgi SNARE, RAB5 effector	—
YKT6	Golgi-localised SNARE, in complex with STX5/GOSR1/BET1L	—
*other genes analysed with an unknown role in Stx trafficking*		
BET1	ER/Golgi-localised SNARE, in complex with STX5/GOSR2/SEC22B	—
COPB2	component of the COPI^l^ vesicle coat	—
GOSR2/GS27	ER/Golgi-localised SNARE, in complex with STX5/BET1/SEC22B	—
SEC22B	ER/Golgi-localised SNARE, in complex with STX18/BNIP1/SEC22B or STX5/GOSR2/BET1	—
STX18	ER-localised SNARE, in complex with BNIP1/USE1/SEC22B	—

^a^'–', neither part of cluster i nor ii.

^b^CCT, coiled-coil tether.

^c^COG, conserved oligomeric complex.

^d^MTC, multisubunit tethering complex.

^e^GAP, GTPase-activating protein.

^f^CORVET, class C core vacuole/endosome tethering.

^g^HOPS, homotypic fusion and protein sorting.

^h^GEF, guanine nucleotide exchange factor.

^i^ESCRT, endosomal sorting complex required for transport.

^j^TRAPP, transport protein particle.

^k^NRZ, NAG-RINT1-ZW10.

^l^COPI, coat protein I/coatomer.

**Figure 5:**
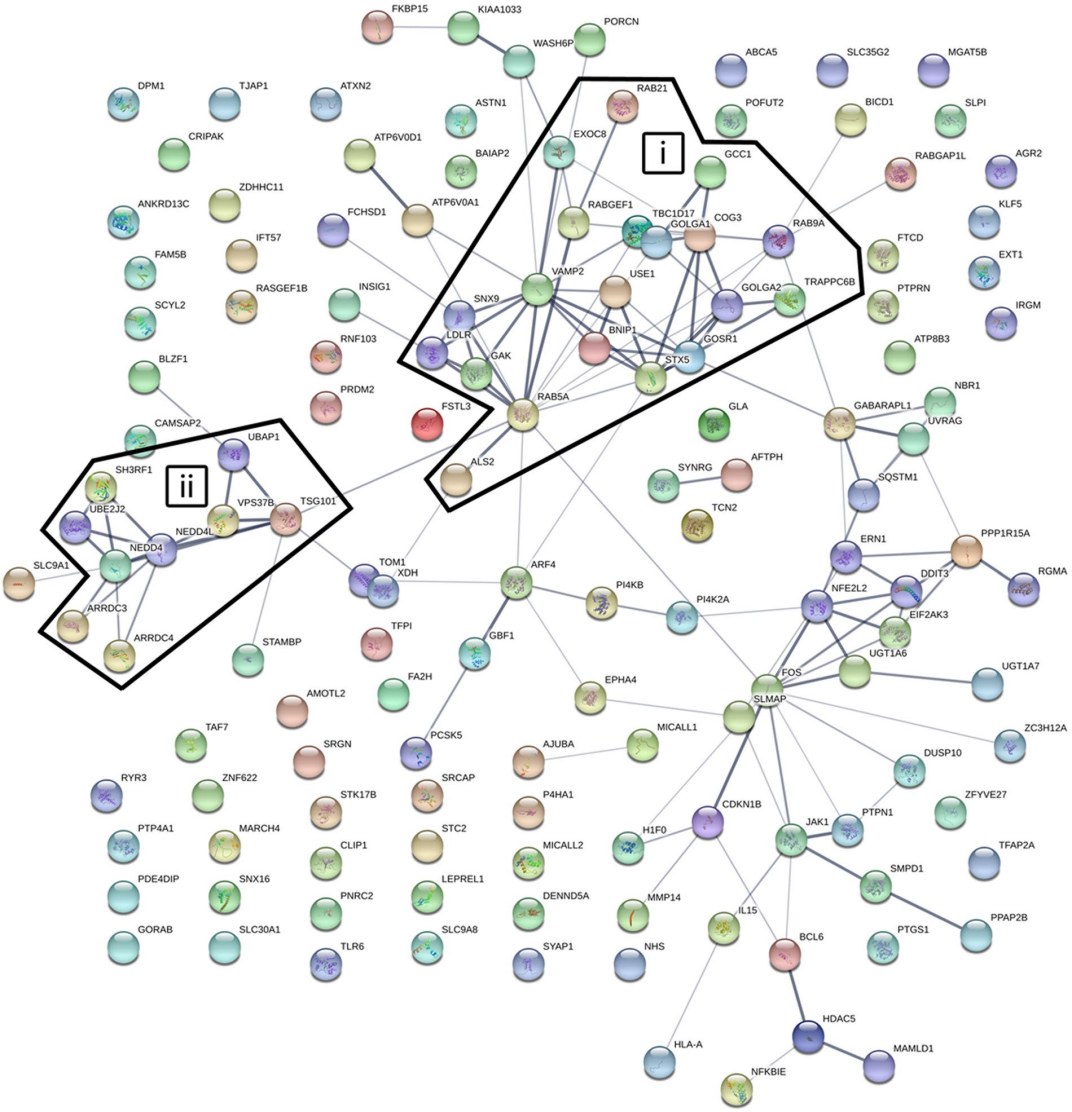
Interaction network of selected genes with higher expression in ACHN versus Caki-2 cells upon Stx2a exposure. A total of 143 genes with enhanced expression, listed in Table S3, was selected from the top 2,000 list of differently expressed genes of ACHN versus Caki-2 cells. STRING database (confidence view) was used for the visualisation of the interaction of genes located at the endosomal system, the Golgi network, or the ER including TGN, *cis*-Golgi network, and ER-Golgi intermediate compartment according to Gene Ontology (with VPS11 from Table S3 missing in the figure due to its absence from STRING). Borders delineate the two clusters i and ii being interconnected by an edge confidence of ≥0.7, from which genes were subsequently analysed.

For this purpose, we first extracted all genes exhibiting a higher expression in ACHN versus Caki-2 cells from the top 2,000 collection that localize in/at the endosomal system, the Golgi network or the ER, including the TGN, the *cis*-Golgi network, and the ER-Golgi intermediate compartment according to Gene Ontology terms (Table S3). Hereafter, we visualised the interaction of these 143 genes using the STRING database (Fig. 5). This interaction network shows several clusters, with one cluster (i) predominantly containing factors involved in intracellular retrograde trafficking, supporting our previous assumption. Another cluster (ii) largely contains factors of the ESCRT (endosomal sorting complexes required for transport) pathway, which also contributes to intracellular trafficking. Importantly, the central node that connects both of the clusters is the gene RAB5A. Hence, by performing interaction analysis of selected differentially expressed genes in ACHN versus Caki-2 cells, we determined distinct clusters containing genes participating in the retrograde trafficking pathway.

### RNAseq highlights that certain trafficking genes in ACHN cells could be silenced to inhibit Stx cytotoxic action

As the ACHN RNAseq data showed a clustering of factors related to intracellular retrograde trafficking (Fig. 5), this indicates that the retro-routing of Stx to its subcellular targets plays a crucial role in ACHN cells’ high sensitivity to Stx2a compared to that of Caki-2 cells. To further explore the RNAseq data and to test the physiological relevance of the host factors identified, we applied RNAi with small interfering RNAs (siRNAs) to ACHN cells. The rationale is that by knocking down specific cellular targets, we should be able to make ACHN cells insensitive to the cytotoxic action of Stx2a (‘refractory effect’).

**Figure 6:**
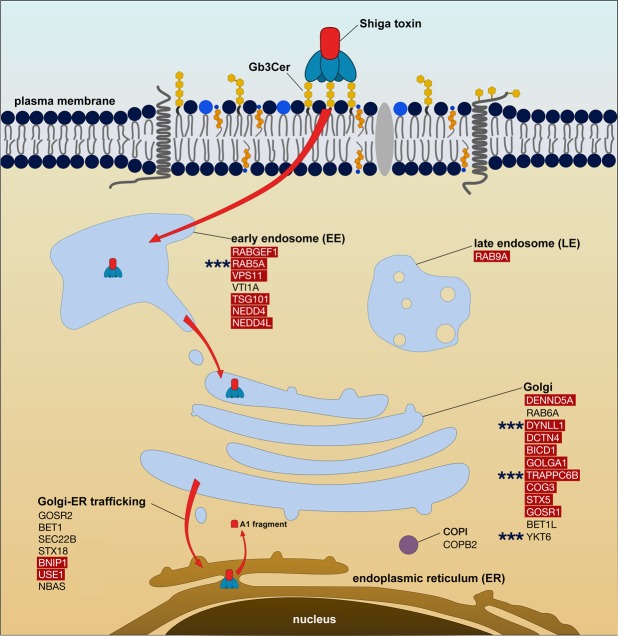
Intracellular trafficking of Stx and cellular targets silenced by RNAi. Depicted is the common trafficking route of Stx upon attachment to its receptor Gb3Cer in the vicinity of sphingomyelin (bright blue circle with grey tail) and cholesterol (orange structure with small blue head). After internalisation, retrograde transport to the EE and then through the Golgi apparatus to the ER, and processing of the Stx A subunit, the A1 fragment is retrotranslocated to the cytosol and exerts its cytotoxic function on ribosomes. Indicated are host genes of ACHN cells targeted with siRNAs prior to Stx2a exposure. Highlighted in red are those genes that exhibited higher expression in ACHN compared to Caki-2 cells with regard to RNAseq data. Asterisks denote those targets, where an siRNA-mediated knockdown resulted in most substantial refractiveness to Stx2a-caused cell injury of ACHN cells (see also Fig. 7).

To this end, we selected a set of targets along Stx’s retrograde trafficking route, i.e. mainly components of the endosomal system, the Golgi apparatus and the ER. The subcellular localisation of the targets is portrayed in Fig. 6, and additional explanations regarding their biological role are provided in Table 1 (also see Table S3 and for corresponding references see Table S2). These targets mostly comprise genes from the top 2,000 list of differentially expressed genes. However, we also included targets not present in this list that either have a well-documented role in Stx trafficking, thus serving as controls, or are interaction partners of one of the other analysed targets. Additionally, because Stx trafficking was shown to be independent of coat protein/coatomer I (COPI) vesicles^33,34^, we included the COPI subunit COPB2 to serve as a negative control.

The success of the siRNA silencing experiments in ACHN cells was shown by RT-PCR, whereby the knockdowns resulted in a significant decrease in mRNA down to 5% to 65% in range in comparison to the negative control siRNA (NC), with a median of 23% (Fig. S4a). To disclose a possible harmful effect of the siRNA treatment itself, the extent of cytotoxicity due to individual siRNAs as well as mixtures of siRNAs for various targets was determined (Fig. S4b). Though transfection *per se* (see mock- and NC-transfected samples versus untransfected samples) had a modest effect on ACHN cell viability, a statistically significant detrimental effect was only observed for six siRNAs, namely TSG101, NEDD4, RAB9A, BICD1, COPB2, and GOLGA1. However, none of these knockdowns had such a strong effect on viability as the PLK1 cell death control. Moreover, when compared to PLK1, three of the aforementioned targets (RAB9A, BICD1, and GOLGA1) showed statistically significant higher viability (statistical comparison to PLK1 is not included in Fig. S4b). Thus, as these knockdown experiments were successful, and the siRNA transfection had mostly insignificant effects on cell viability, we proceeded to use the knockdown approach for Stx2a inhibition experiments.

### Silencing of RAB5A, TRAPPC6B, and YKT6 abrogates Stx2a intoxication of ACHN cells

**Figure 7:**
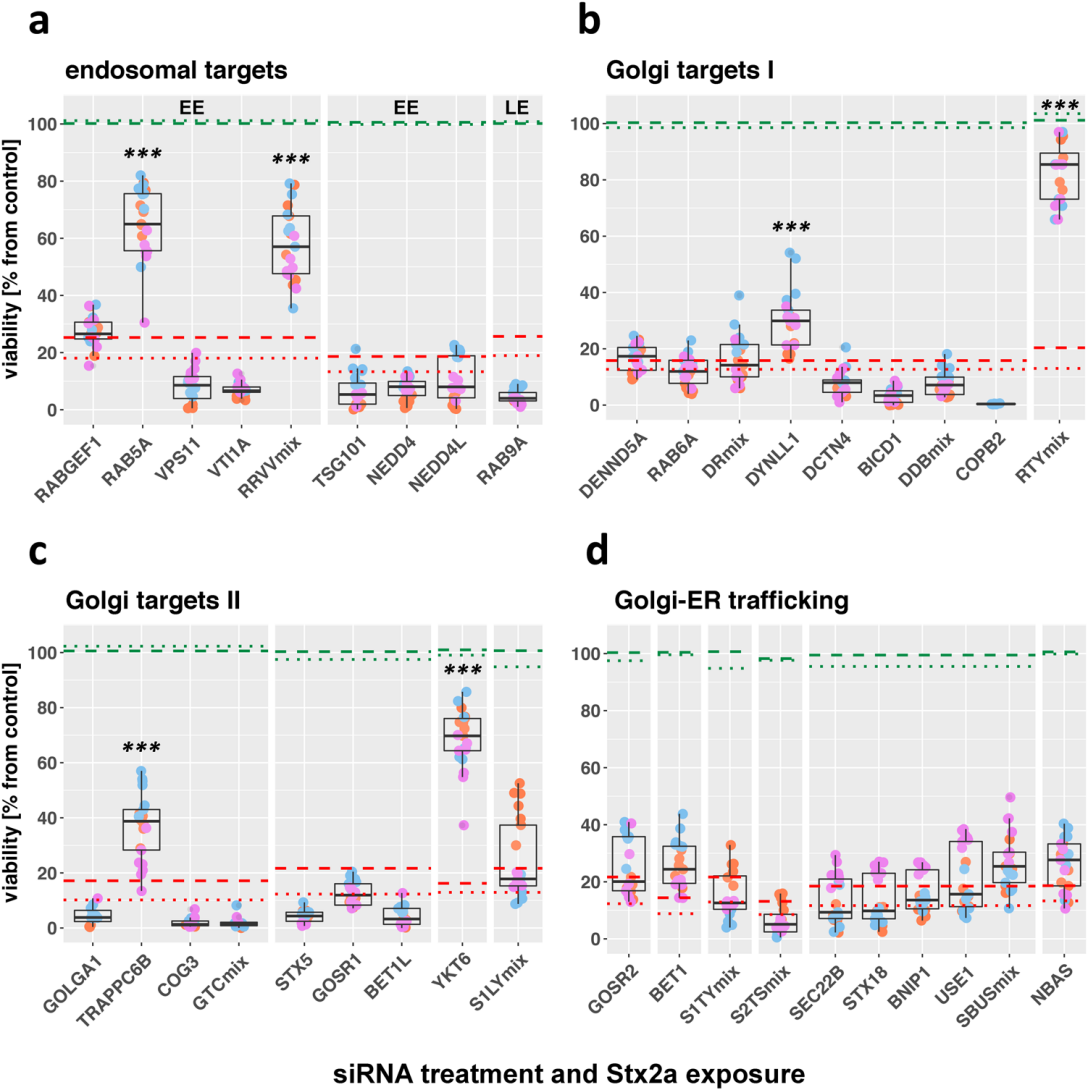
Survival of ACHN cells upon application of siRNA prior to 72 h Stx2a exposure. ACHN cells were not transfected or were reverse transfected with a scrambled NC or the indicated siRNA(s) directed towards the following targets: endosomal targets (**a**), two different sets of Golgi-targets (**b**,**c**), and Golgi-ER trafficking targets (**d**). Then, cells were incubated without or with Stx2a for 72 h. Cell viability upon application of siRNA and Stx2a is depicted as the percentage related to untreated cells alongside with boxplots. Each biological replicate (*n* = 3) shown in different colours (orange, magenta, and blue) was technically replicated a further seven times. Median values from 21 measurements each of transfected and non-transfected controls are shown as dotted and dashed lines, respectively. Approaches without Stx2a are displayed in green and those with Stx2a in red. Measured values of non-transfected cells without Stx2a were set as 100% viability. Spaces indicate that data was taken from different sets of microtiter plates. For separate plots depicting all controls refer to Fig. S5a. Statistical analysis is based on linear models. Asterisks above boxplots indicate levels of significance in relation to NC- and Stx2a-exposed cells (red, dotted line) and are only given for knockdowns with at least 15% difference to control and 30% viability (i.e. two-fold difference): ***p < 0.001. Abbreviations: mix, mixture; RRVVmix, RABGEF1, RAB5A, VPS11, and VTI1A; DRmix, DENND5A and RAB6A; DDBmix, DYNLL1, DCTN4, and BICD1; RTYmix, RAB5A, TRAPPC6B, and YKT6; GTCmix, GOLGA1, TRAPPC6B, and COG3; S1LYmix, STX5, GOSR1, BET1L, and YKT6; S1TYmix, STX5, GOSR1, BET1, and YKT6; S2TSmix, STX5, GOSR2, BET1, and SEC22B; SBUSmix, STX18, BNIP1, USE1, and SEC22B.

After ACHN cells were treated with siRNA, they were exposed to Stx2a for 72 h followed by viability measurements (Fig. 7). The cell viability of Stx2a-exposed but otherwise only NC- or untransfected cells (controls) ranged from 15 to 25%. Many of the individual or mixed knockdowns exhibited a low but significant increase or decrease in viability compared to the controls (see Table S5). However, to focus on biologically relevant refractory effects, we only considered targets the knockdown of which resulted in at least doubled cell viability (30%) compared to NC- and Stx2a-exposed controls (15%).

In the endosomal group of targets (Fig. 7a), knockdown of RAB5A as well as that of a mixture of RAB5A with its guanine nucleotide exchange factor (GEF), RABGEF1, and two of its effectors, VPS11 and VTI1A (RRVVmix), elicited a relevant increase in cell survival up to 62.7% and 55.3%, respectively. As targeting RAB5A alone had a slightly higher refractory effect than using the RRVVmix, no other components in the RRVVmix seem to influence Stx2a sensitivity.

In the RAB6A-centered Golgi group of targets (I, Fig. 7b), none of the tested individual or combined knockdowns (except RTYmix, see below) caused a relevant refractory effect except for a slight beneficial effect indicated by 31.7% cell survival when targeting DYNLL1. However, unlike the RAB5A mixtures, mixtures of DYNLL1 with other microtubule-associated factors, namely DCTN4 and BICD1 (DDBmix), did not produce an increase in cell survival. No effect was seen for knocking down COPB2, which was expected as this was a negative control (see above).

In the other group of Golgi targets (II, Fig. 7c), knockdown of TRAPPC6B, as well as YKT6, increased the viability relevantly up to 37.5% and 68.6%, respectively. However, similar to DYNLL1, mixtures of TRAPPC6B with other tethering factors, namely GOLGA1 and COG3 (GTCmix), and mixtures of YKT6 with other SNAREs, namely STX5, GOSR1, and BET1L in the S1LYmix (or BET1 in the S1TYmix, as in Fig. 7d), did not show a comparable increase in cell viability.

Regarding anterograde ER-Golgi trafficking-related SNAREs (a process unknown to participate in Stx trafficking), neither GOSR2 or BET1 alone nor combinations of them with other SNAREs (S1TYmix and S2TSmix) showed a relevant refractory effect (Fig. 7d). Lastly, no beneficial effect was detected for the remainder of the individual or mixed retrograde Golgi-ER trafficking components (Fig. 7d).

Finally, using a mixture that knocked down all of the targets that had prominent benefits for cell survival, namely RAB5A, TRAPPC6B, and YKT6 (RTYmix), we found a cumulative refractory effect, whereby silencing increased viability up to 81.3% (Fig. 7b); hence, this mixture almost completely prevented Stx2a toxicity.

**Figure 8:**
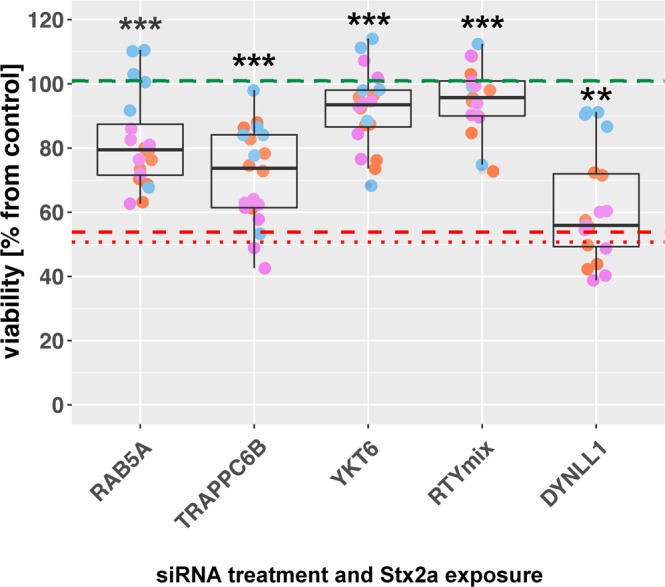
Survival of ACHN cells upon application of siRNA for selected most promising targets prior to 48 h Stx2a exposure. Experimental details correspond to those of Fig. 7 except that Stx2a exposure was shortened to 48 h. Depicted are three biological replicates (*n* = 3) each performed in septuplicate. Median values from 21 measurements each of transfected and non-transfected controls are shown as dotted and dashed lines, respectively. Approaches without Stx2a are displayed in green and those with Stx2a in red. Results of non-transfected cells without Stx2a were set as 100% viability. For separate plots depicting all controls, see Fig. S5b. Statistical analysis was performed against NC- and Stx2a-treated cells with linear models: **p < 0.01; ***p < 0.001.

To test whether the duration of the Stx2a challenge influences the refractory effect of selected individual or combined siRNA knockdowns, we reduced the time of Stx2a exposure from 72 h to 48 h (Fig. 8), another common incubation time for Stx cytotoxicity assays^35^. The reduced exposure time of 48 h resulted in an overall elevated viability in comparison to 72 h, which was then further increased from 52.2% of NC-transfected and Stx2a-exposed cells by targeting single components to 82.2% with RAB5A, 71.2% with TRAPPC6B, or 92.4% with YKT6 (though the beneficial effect of DYNLL1 was less pronounced, with 64.3%). Remarkably, knockdown with the RTYmix reached 99.5%, producing virtually a full inhibition of the Stx2a intoxication.

Taken together, by using RNAi in ACHN cells aimed at a panel of targets involved in intracellular trafficking and then exposing them to Stx2a, we were able to identify three major targets as being pivotal in determining the differing Stx susceptibilities of ACHN and Caki-2 cells, i.e. RAB5A, TRAPPC6B, and YKT6. Two of these, RAB5A and TRAPPC6B, constitute completely novel and unprecedented host factors in Stx biology. Silencing these two factors in combination with YKT6 reverted the devastating cytotoxic action of Stx.

## Discussion

As the renal epithelium is an important Stx target during HUS^11^, new research is needed to elucidate in detail the interactions of Stx with these cells. Here, we investigated two renal epithelial cell lines, ACHN and Caki-2, which have strikingly opposing responses to Stx intoxication. By understanding this difference in more detail, our study offers an opportunity to protect the renal epithelium *in vivo*.

While previous studies have determined, using TLC or flow cytometry, that Stx was able to access the receptor Gb3Cer on ACHN cells^36,37^, such studies had not been performed for Caki-2 cells. To address this, here we used immunofluorescence microscopy to show that Stx2a could access ACHN as well as Caki-2 cells (Fig. S2). Furthermore, using TLC overlay assays, we detected identical Gb3Cer binding profiles of Stx2a for both ACHN and Caki-2 cells (Fig. 2b). Additionally, by providing specific structural data, our work supplements previous findings that Gb3Cer is definitively present on ACHN cells, which was found by employing antibodies either by flow cytometry^30,36^ or by TLC^37^. Moreover, it clarifies the previous finding from a study using flow cytometry that Gb3Cer is only marginally present on Caki-2 cells^30^. Here, for the first time, we present high-resolution mass spectrometry data on various Gb3Cer lipoforms shared by both ACHN and Caki-2 cells, unequivocally showing that Gb3Cer is present on both cell types (Fig. 2c). In sum, while these findings are in perfect agreement with early studies on ACHN cells that described their high sensitivity to Stx^29^, which is also what we found here (Fig. 1), these data do not explain why Caki-2 cells are refractory to Stx, as shown before^30^ and in our study (Fig. 1). Indeed, the Stx2a binding to ACHN and Caki-2 cells differs to some extent (Fig. S2), which might result in the contrasting sensitivity and definitely needs further analysis in the future. However, the virtually equivalent Stx receptor profiles and the fact that single Stx molecules suffice to kill cells^32^ prompted us to instead apply a high-throughput approach for transcriptomic profiling using RNAseq to unravel why apparently similar renal epithelial cells have such opposite sensitivities to the toxin.

Transcriptomic profiling after Stx challenge has been previously executed by microarray analyses. These studies used human umbilical vein endothelial cells^38^, human macrophage-like THP-1 cells^39^, and human dermal neonatal microvascular endothelial cells^40^, and differential expression was found for 38, 36, and 369 genes, respectively. Here, our RNAseq data suggest that the cellular response to Stx exposure encompasses a wider set of affected genes (Fig. 3), which may have been difficult to observe in previous studies, as microarray techniques are less sensitive than RNAseq.

When comparing the top 20 genes we found to be most notably affected by Stx (Fig. 4) with the genes found in the previous microarray studies, it becomes obvious that in all the studies five out of the eight genes common between ACHN and Caki-2 cells are upregulated, namely ATF3, DUSP1, EGR1, TRAF1, and TNFAIP3. Likewise, CXCL8/IL8, which is in the top 20 genes in Caki-2 cells but is upregulated in ACHN cells as well, was found to be affected by Stx in the aforementioned microarray studies. As most of these genes are related to inflammation, this strongly suggests that upon Stx intoxication these genes function in a cell type-independent inflammation-related way. Nevertheless, as these similarities do not disclose why ACHN cells are more sensitive to Stx than Caki-2 cells, we focused on differences that arose in response to a toxin challenge using time series analysis; this helped narrow our approach down to differences in trafficking pathways (Figs. 5 and 6).

Most importantly, the key targets we identified with regard to Stx intracellular trafficking, RAB5A and TRAPPC6B, are completely unprecedented. This underlines the power of our approach using RNAseq in the first place as it successfully recognised novel crucial factors in Stx biology which were missed in Stx trafficking research until now. Though the early steps of Stx trafficking, where the EE is the intracellular starting point, have been investigated in great detail^21,22,24^, no study has previously demonstrated the direct involvement of RAB5A, the master regulator of trafficking at the EE; in fact, one study even showed that Stx transport to the Golgi apparatus was independent of RAB5A^41^. In contrast, here, for the first time, we revealed that RAB5A crucially participates in Stx trafficking in ACHN cells (Figs. 7a and 8), which is reasonable regarding its biological function. This discrepancy from previous work can be explained by the use of different cell lines: we used renal epithelial ACHN cells, whereas previous work used renal fibroblast COS-7 and cervical epithelial HeLa cells. Additionally, other methodology might contribute to this discrepancy as well, i.e. we used Stx cytotoxicity analyses, whereas previous work used Stx uptake assays and microscopy for readout, which does not directly assess cell viability. Together, these factors could have caused previous work to overlook RAB5A’s essential role in Stx toxicity. This is corroborated by a recent genome-wide CRISPR/Cas screen performed in urinary bladder epithelial 5637 cells that used cytotoxicity as readout; it provided RAB5A as a hit^42^.

Three recent genome-wide screens for Stx cytotoxicity have been conducted, one using RNAi^43^ and two using CRISPR/Cas^42,44^. In comparison to these studies, our approach, besides applying RNAseq for the first time to explore Stx-host interaction, has substantial advantages. First, in contrast to disease-unrelated cell types, we used actual pathogenesis-involved cell types. Second, our approach of using RNAseq first, in contrast to using RNAi directly from the beginning or using CRISPR/Cas, most likely helped us identify host factors that otherwise would appear as false negatives or only as minor hits, e.g. due to their essentiality; in fact, this might be why RAB5A was not identified earlier. Thus, our approach to combine RNAseq with RNAi merges the advantages of both techniques. Moreover, RNAseq, suitable for short incubation intervals, can be especially helpful in uncovering the factors at play in the early responses to Stx, which are potentially masked by RNAi or CRISPR/Cas, as these approaches are usually applied on the scale of days. Our approach also has certain limitations: in case of highly stressed conditions upon Stx2a exposure, significantly upregulated genes are not only the ‘targets’ for Stx but also the ‘consequences’ of Stx cytotoxic action. However, examining the functional relationship between upregulated genes allows for selecting potential candidates for RNAi experiments, which are not surprisingly trafficking genes. In this case, RNAseq is an approach that can point to particular genes in the trafficking pathway, worth selecting as cell type-specific candidates for RNAi. In the future, this approach should be tested using primary cells of kidney and brain with reduced Stx incubation time.

Altogether, our study revealed, and future studies using our approach will help to reveal, crucial factors in Stx biology that can potentially be exploited for novel therapeutic applications. This is of outstanding importance, as non-bacterial targets (those that do not require antibiotics) are desperately needed to treat the life-threatening conditions caused by Stx.

## Methods

### Cell culture and cytotoxicity assays

Human kidney epithelial cell lines were obtained from the American Type Culture Collection (ATCC, Manassas, VA). ACHN (ATCC, CRL-1611) and Caki-2 (ATCC, HTB-47) epithelial cell lines were adapted to and cultivated at 37 °C in a humidified 5% CO_2_ atmosphere in OptiPRO^TM^ serum-free medium (#12309–019, Gibco Life Technologies Corporation, Paisley, UK), which excluded uptake and detection of exogenous GSLs from serum. Cell medium was additionally supplemented with 4 mM L-glutamine. ACHN and Caki-2 cells were cultured as needed in 25–175 cm^2^ tissue culture flasks (Greiner Bio-One, Frickenhausen, Germany) and passaged every 2–3 days using 0.25% Trypsin-EDTA (#25200, Invitrogen, Karlsruhe, Germany).

Stx2a was affinity-purified from the Stx2a-containing supernatant of *E. coli* strain 03–0616 (O111:H^−^) as previously described^45^ and proteins in the SDS-PAGE were stained with the Quick Coomassie Stain (35081.01, Serva, Heidelberg, Germany) using the Precision Plus Protein Dual Xtra Prestained Protein Standard as reference (5 µL, 1610377, Bio-Rad, Munich, Germany) (Fig. S1). Stx2a preparations were free of bacterial endotoxins as measured by the Pierce LAL Chromogenic Endotoxin Quantification Kit (#88282, Thermo Fisher Scientific, Dreieich, Germany).

The cytotoxicity of Stx2a was assessed with the crystal violet assay as previously described using the indicated concentrations (Fig. 1) or 40 pg/mL (Figs. 7 and 8). For RNAseq, ACHN and Caki-2 cells were exposed to Stx2a for 4 h and 8 h at a concentration of 0.4 µg/mL or were left unexposed (control condition). Three biological replicates were prepared per cell line and condition.

### Lipid reference, antibodies, and fluorophores

Neutral GSLs from human erythrocytes containing the Stx receptor GSLs Gb3Cer and Gb4Cer served as a positive control for orcinol staining and Stx2a TLC overlay assays (Fig. 2a,b)^46^.

Monoclonal mouse IgG anti-Stx2 antibody (clone VT 135/6-B9, 2.75 mg/mL, SIFIN GmbH, Berlin, Germany) was used for TLC overlay assays (Fig. 2b) and for immunofluorescence imaging (Fig. S2). Highly cross-absorbed Alexa Fluor 488 goat anti-mouse IgG antibody (#A11029, Thermo Fisher Scientific) was used for immunofluorescence imaging (Fig. S2). Secondary alkaline phosphatase (AP)-conjugated affinity-purified polyclonal goat anti-mouse IgG antibody (code 115–055–003, Dianova, Hamburg, Germany) was used for TLC overlay assays (Fig. 2b). WGA conjugated to Alexa Fluor 647 (#W32466, Thermo Fisher Scientific), goat anti-mouse IgG antibody conjugated to Alexa Fluor 488 (#A11029, Thermo Fisher Scientific), and 4′,6-diamidino-2-phenylindole (DAPI, #D9542, Sigma-Aldrich, Taufkirchen, Germany) were used for immunofluorescence imaging (Fig. S2).

### GSL isolation and TLC overlay assays

GSLs were extracted from confluently grown total cells with methanol and various chloroform/methanol mixtures. Coextracted glycerophospholipids and triglycerides were removed by mild saponification. Neutral GSLs were separated from acidic GSLs by anion-exchange column chromatography using DEAE-Sepharose CL-6B (GE Healthcare, Munich, Germany) according to standard procedures^46^ and finally dissolved in chloroform/methanol (2/1, v/v). Purified neutral GSLs were applied to high-performance TLC plates precoated with silica gel 60 (size: 10 cm × 10 cm; thickness: 0.2 mm; #1.05633.0001, Merck, Darmstadt, Germany) with an automated sample applicator (Linomat 5, CAMAG, Muttenz, Switzerland). Subsequently, neutral GSLs were separated in chloroform/methanol/water (120/70/17, v/v/v) and stained with orcinol or subjected to TLC overlay assays as previously described^46,47^. Shortly, Gb3Cer was detected with Stx2a combined with a primary anti-Stx2 and an AP-conjugated secondary antibody.

### Mass spectrometry of Gb3Cer

MS^1^ and MS^2^ analysis of Gb3Cer was performed using a SYNAPT G2-S mass spectrometer (Waters, Manchester, UK) equipped with a Z-spray source as previously described^45,47^. Purified neutral GSLs from ACHN and Caki-2 cells were analysed in the positive ion sensitivity mode. Structures of individual GSLs were detected as singly charged monosodiated [M + Na]^+^ ions and structures were deduced from CID spectra.

### Immunofluorescence microscopy

ACHN and Caki-2 cells were seeded at a concentration of 1 × 10^5^ cells/mL (final volume 0.2 mL)  in 8-well polystyrene chamber slides (#177445, Thermo Fisher Scientific) and cultured for two days until ~80–90% of confluence. To facilitate the assessment of the subcellular distribution of the toxin receptors and for the purposes of the fluorescent signal normalisation, the surface of the plasma membrane of the cells was pre-stained with WGA-Alexa Fluor 647 for 15 min prior to fixation, quenching, and incubation with the toxin. Afterwards, cells were fixed with 3.7% paraformaldehyde (Merck) for 30 min, quenched with 0.2 M glycine, pH 7.2 (Carl Roth, Karlsruhe, Germany) for 15 min, and incubated with Stx2a (0.5 μg/mL) for 1 h. Stx2a-exposed samples were then incubated with anti-Stx2a antibody in 1:500 dilution with 1% BSA at 4 °C overnight followed by Alexa Fluor 488-coupled antibody for 1 h. Nuclear DNA was stained with DAPI for 10 min. Finally, slides were mounted with Immunoselect Antifading Mounting Medium (#SCR-038447, Dianova).

The Stx2a immunostained preparations (3 biological replicates) were accompanied by mock-immunostained samples (2 biological replicates) which were treated identically to the experimental samples with the omission of Stx2a. Immunofluorescence imaging was performed with Leica SP8 confocal laser scanning microscope equipped with an HC PL Apo CS2 63x NA 1.4 oil immersion objective and HyD detectors for photon counting (Leica, Wetzlar, Germany). Collected confocal stacks are presented as maximum intensity XY projections and transverse XZ and YZ sections. To quantify the difference in Stx2a binding between ACHN and Caki-2 cells we first established the background fluorescence level which was obtained by staining both cell lines with WGA and both primary and secondary antibodies without adding the toxin. For this, the cumulative unspecific antibody signal from each stack obtained from the mock-immunostained samples (*n* = 31 and *n* = 51 for ACHN and Caki-2 cells, respectively) was normalised against the cumulative WGA signal of each respective stack (antibody/WGA ratio). Here, the WGA signal provides a way to approximate the total cell surface available for toxin binding. Such a normalisation ensures that the result is independent of the cell geometry, the number of cells within the field of view, and the stack dimensions. The fact that the baseline antibody/WGA ratio is nearly consistent between the two cell lines (0.32 versus 0.35 for ACHN and Caki-2 cells, respectively) confirms the utility of such an approach. Once the baseline antibody/WGA ratio has been established, the fluorescence signal in the presence of the toxin was scored as the difference between this signal and the background for each respective cell line (*n* = 115 and *n* = 92 for ACHN and Caki-2 cells, respectively). The imaging data were processed and analysed with ImageJ 1.51 h (National Institutes of Health, USA).

### RNA isolation and RNA sequencing

Total RNA was purified from ACHN and Caki-2 cells using the TRIzol Reagent (Thermo Fisher Scientific) following the manufacturer’s instructions. We performed a quality check and quantification of total RNA with the Bioanalyzer RNA 6000 Nano Kit (Agilent Technologies). mRNA-Seq libraries were prepared from 1 μg of total RNA (RIN > 9.6) using the Agilent SureSelect Strand-Specific RNA Library Prep following the manufacturer’s instructions. 36 bp single-end sequencing was conducted using a HiSeq 3000 machine (Illumina) and the sequence summary is presented in Table S1.

### Bioinformatic analysis of RNAseq data and visualisation

The quality of the RNAseq reads was assessed with FastQC software (v0.11.5, http://www.bioinformatics.babraham.ac.uk/projects/fastqc). RNAseq reads were mapped to human reference genome version GRCh38.87 with the TopHat2 aligner (v2.0.13)^48^. Mapped reads (in bam format) were processed with SAMtools (v1.3.1, http://samtools.sourceforge.net): bam files were sorted by query name and multiple alignments were removed. Unique alignments in bam format were subsequently used in gene-level exploratory analysis and differential expression using R (v.3.4.1)^49^ and DESeq2 package (v1.16.1) following instructions of Love and co-authors^50^. Briefly, gene models were defined using the pre-built transcript database TxDb.Hsapiens.UCSC.hg38.knownGene and count matrices were generated with the summarizeOverlaps function. Finally, a DESeqDataSet object was constructed and genes with counts < 1 were filtered out. The regularised-logarithm transformation (rlog) was applied prior to PCA to stabilize the variance across the mean. PCA was performed to visualize the variation between the groups (cells with Stx2a treatment and without) and within the groups (biological replicates). MA plots (minus/average; mean-difference plots) were constructed to visualize the changes induced by Stx2a treatment. Time-series analysis was performed to find genes most differentially expressed in ACHN and Caki-2 cells in response to Stx2a treatment, taking into account different gene expression in these cells under control conditions (no Stx2a). Figures outside the DESeq2 package were created using R package ggplot2^51^ and Adobe Photoshop CC 2017.

### RNA interference via reverse transfection

For RNAi individual siRNAs were ordered from Qiagen (Hilden, Germany) with the scrambled AllStars siRNA serving as NC. For a full list see Table S4. First, the amount for an end concentration of 5 nM per siRNA was spotted onto 96-well plates (Greiner Bio-One). A mixture of all siRNAs targeting a gene was applied where available. Next, a mixture of the transfection reagent HiPerFect (Qiagen) and ACHN cell culture medium was applied and incubated for 10 min at RT. Then, 9,000 cells per well in medium were added and incubated for 48 h at 37 °C in a humidified atmosphere containing 5% CO_2_. If subsequent Stx2a exposure was performed, the toxin was applied for another 48 h or 72 h. Cytotoxicity measurement with knockdowns alone or with additional Stx2a treatment was executed as described above.

### RNA isolation for RT-PCR

After reverse transfection with different siRNAs, total RNA of cells was extracted using the RNeasy Mini Kit following the manufacturer’s instructions (Qiagen). Homogenisation during extraction was performed using Qiagen QIAshredder columns. Next, RNA was digested with the Turbo DNA-free Kit (Thermo Fisher Scientific) following the manufacturer’s instructions. Then, 2 ng of DNase-digested RNA was used for one-step RT-PCR with the QuantiTect SYBR Green RT-PCR Kit (Qiagen). The housekeeping gene *GAPDH* was selected as reference^52^. The primers used for the control of individual target knockdowns were selected employing the PrimerBank database^53,54^. Primers for those as well as *GAPDH* are listed in Table S4. The primer concentration was 500 nM each and for each primer pair the primer efficiency was determined. RT-PCR was performed in triplicates in 96-well plates in a CFX96 machine (Bio-Rad). Relative expression was determined using the 2^−ΔCt^ method^55,56^ with *GAPDH* expression set as 100%. Results were plotted using ggplot2.

### Statistical analysis

Statistical analysis was performed in R (v.3.4.1)^49^. Cytotoxicity assays with Stx2a (Fig. 1) or siRNA only (Fig. S4b) as well as Stx2a assays with siRNA pre-treatment (Figs. 7 and 8) were analysed using mixed effects models in R with the nlme package^57^. Briefly, in case of mixed effects models for cytotoxicity assays (Fig. 1), treatment with Stx2a (‘treatment’) was set as a fixed effect and biological replicates were set as random factors (random = ~1|replicate). The model also took into account differences in variances between treatment groups (weights = varIdent (form = ~1|treatment)):

1 $$\begin{array}{c}{\rm{nlme}}\,({\rm{viability}}\sim {\rm{treatment}},\,{\rm{random}}=\sim \,1|{\rm{replicate}},\\ {\rm{weights}}={\rm{varIdent}}\,({\rm{form}}\sim 1|{\rm{treatment}}),\,{\rm{method}}={\prime\prime} {\rm{REML}}{\prime\prime} )\end{array}$$

The same approach was applied for cytotoxicity assays with siRNAs only (Fig. S4b) and Stx2a assays with siRNA pre-treatment (Figs. 7 and 8) with the difference that siRNAs were set as fixed effects instead of Stx2a treatment. Validations of the statistical models for Figs. 1, S4b, 7 and 8 are provided in Fig. S6a–d, respectively. For the summaries of the statistical models used for data from Figs. 1, S4b, 7 and 8 see Table S5.

ANOVA was performed in order to estimate if there are differences between siRNA knockdowns in ACHN cells. Multiple comparisons of siRNA knockdowns were performed using *t*-tests (*n* = 3) and *p*-values were adjusted for multiple comparisons using the Holm method (Fig. S4b).

Time-series analysis to reveal cell-specific responses to Stx2a exposure was performed in the DESeq2 package as described by Love *et al*.^58^. Briefly, we modelled the cell difference (ACHN/Caki-2) at time point 0 (control), the difference over 4 h and 8 h of Stx2a exposure (treatment), and any cell-specific difference over time points (the interaction term ‘cell:treatment’):

2 $${\rm{ddsTC}} < -\,{\rm{DESeqDataSet}}\,(\,\sim \,{\rm{cell}}+{\rm{treatment}}+{\rm{cell}}:{\rm{treatment}})$$

Next, we performed a likelihood ratio test (LRT) where cell-specific differences were removed:

3 $${\rm{ddsTC}} < -\,{\rm{DESeq}}\,({\rm{ddsTC}},\,{\rm{test}}\,=\,{\prime} \text{LR}{\rm{T}}{\prime} ,\,{\rm{reduced}}=\sim \,{\rm{cell}}+{\rm{treatment}})$$

The output of this test was a list of genes with small *p*-values that showed cell-specific effect after time point 0. Genes that moved up or down similarly over time were not given priority by this model design.

### Accession code

The RNAseq data are  available at the European Nucleotide Archive (Accession number PRJEB33446). Other relevant data are presented in supplementary materials or will be provided by the corresponding authors upon request.

## Supplementary information

Supplementary material.

Supplementary table S3.
