# Supplementary material for: RAB5A and TRAPPC6B are novel targets for Shiga toxin 2a inactivation in kidney epithelial cells
Source: Sci Rep. 2020 Mar 18;10:4945. doi: 10.1038/s41598-020-59694-w (PMC7080763; doi:10.1038/s41598-020-59694-w)
Supplement: Supplementary file 1 — Supplementary material. [file 41598_2020_59694_MOESM1_ESM.pdf]

RAB5A and TRAPPC6B are novel targets for Shiga toxin 2a inactivation in kidney epithelial cells

Ivan U. Kouzel[1,5,*], Alexander Kehl[1,*], Petya Berger[1], Ivan Liashkovich[2], Daniel Steil[1], Wojciech Makalowski[3], Yutaka Suzuki[4], Gottfried Pohlentz[1], Helge Karch[1], Alexander Mellmann[1], and Johannes Müthing[1]

[1]Institute for Hygiene and National Consulting Laboratory for Hemolytic Uremic Syndrome (HUS), University of Münster, D-48149 Münster, Germany

[2]Institute for Physiology II, University of Münster, D-48149 Münster, Germany

[3]Institute of Bioinformatics, University of Münster, D-48149 Münster, Germany

[4]Department of Computational Biology and Medical Sciences, University of Tokyo, Japan

[5]Present address: Sars International Centre for Marine Molecular Biology, University of Bergen, Norway

*These authors contributed equally to this work.

Correspondence and requests for materials should be addressed to I.U.K. (Ivan.Kouzel@uib.no) or A.K. (alexander.kehl@ukmuenster.de)

Supplementary Material

Supplemental Figures and Tables

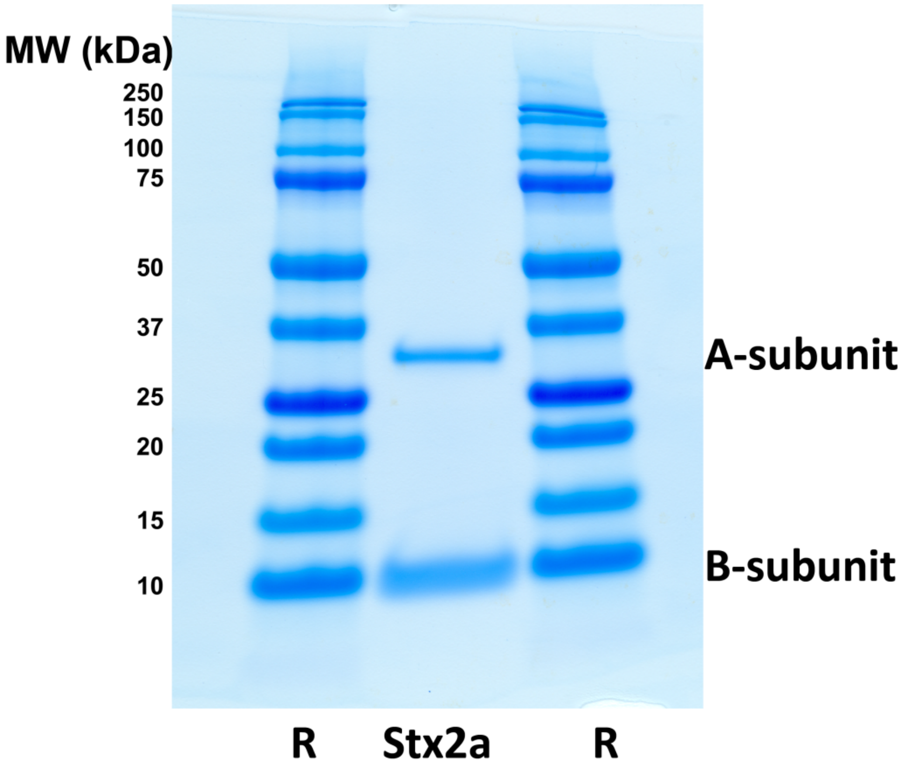

Figure S1. SDS-PAGE of highly affinity-purified Stx2a. This charge was used for cytotoxicity assays of ACHN and Caki-2 cells. The amount of Stx2a applied was 10 µg and proteins were stained with Coomassie blue. R – reference proteins.

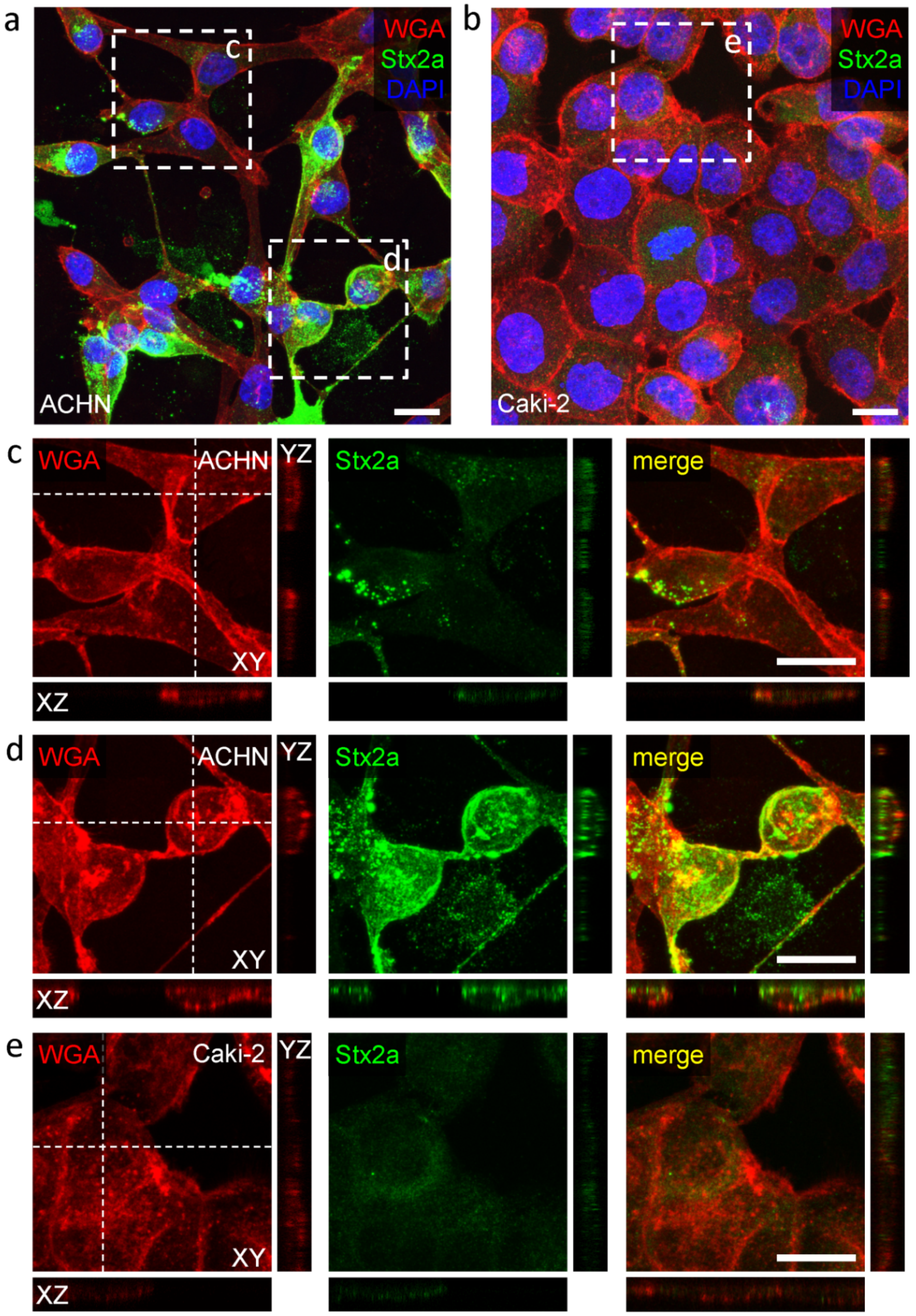

Figure S2. **Fluorescence microscopy imaging of Stx2a binding to ACHN and Caki-2 cells.** ACHN (a) or Caki-2 (b) cells were prestained with Alexa Fluor 647-labelled WGA, incubated with Stx2a, and Stx2a binding was visualised with an Stx2a antibody and a corresponding

Alexa Fluor 488-labelled secondary antibody. The cell nuclei were stained with DAPI. (c-e) Magnifications of the regions delineated by dashed boxes in (a) and (b) depict either two different populations of ACHN cells (c, d) or Caki-2 cells (e), respectively. The white cross-hairs in the WGA panels in (c-e) indicate the locations of the transverse XZ and YZ sections through each of the respective 3D stacks. Scale bars = 20 µm.

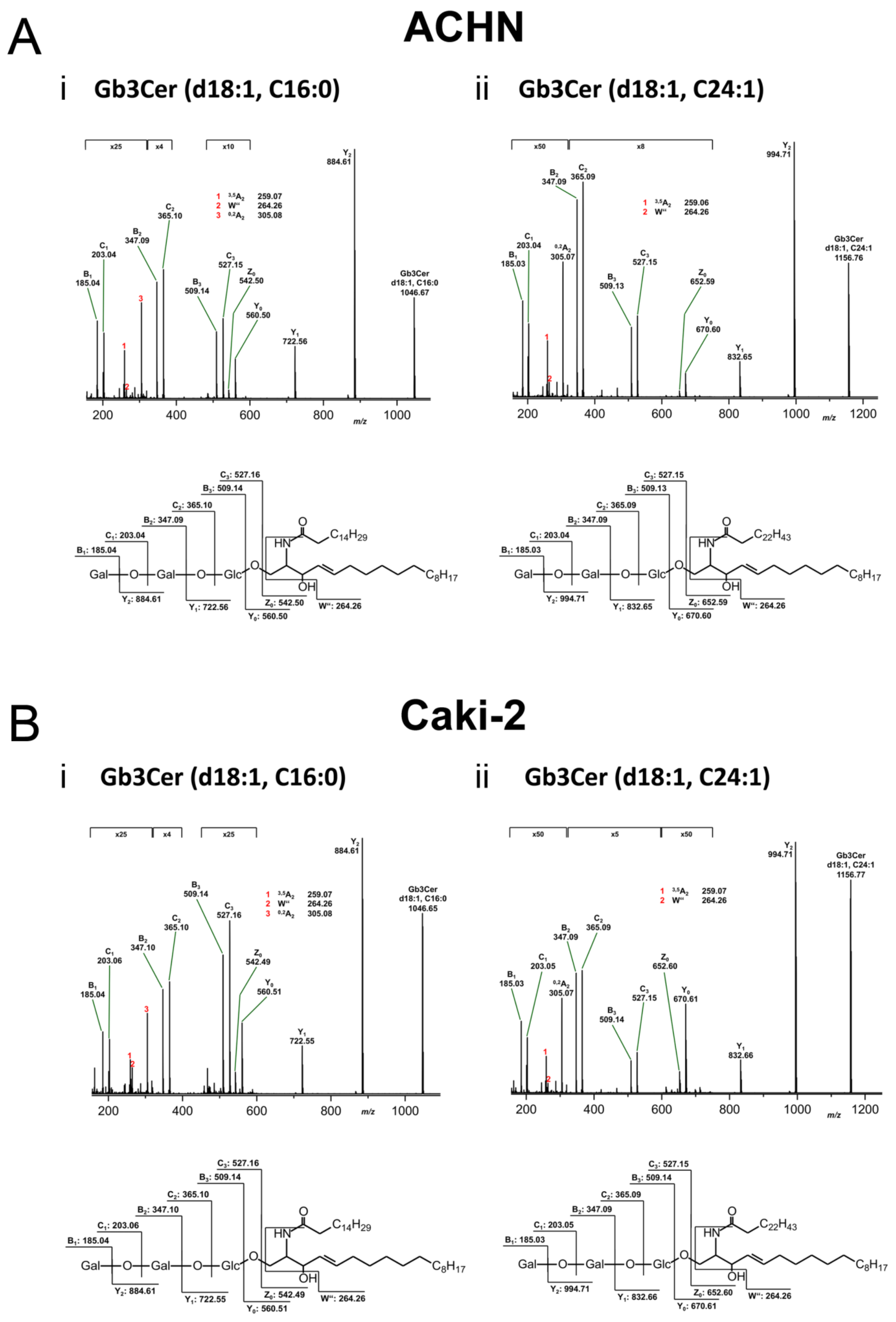
A
ACHN
i Gb3Cer (d18:1, C16:0)
ii Gb3Cer (d18:1, C24:1)
Y2 884.61
Gb3Cer d18:1, C16:0 1046.67
Y2 994.71
Gb3Cer d18:1, C24:1 1156.76
m/z
B
Caki-2
i Gb3Cer (d18:1, C16:0)
ii Gb3Cer (d18:1, C24:1)
Gb3Cer d18:1, C16:0 1046.65
Gb3Cer d18:1, C24:1 1156.77
Gal
Glc
HN
OH

Figure S3. ESI MS^2 spectra and explanatory fragmentation schemes of selected Gb3Cer lipoforms. The singly charged $[M+Na]^+$ precursor ions at *m/z* 1046.67 (A) or 1046.65 (B), respectively, corresponding to Gb3Cer (d18:1, C16:0) (i) and at *m/z* 1156.76 (A) or 1156.77 (B), respectively, corresponding to Gb3Cer (d18:1, C24:1) (ii) were selected from MS^1 spectra of the GSL fraction of ACHN cells (A) or Caki-2 cells (B) for MS^2 analysis (**Error! Reference source not found.**c).

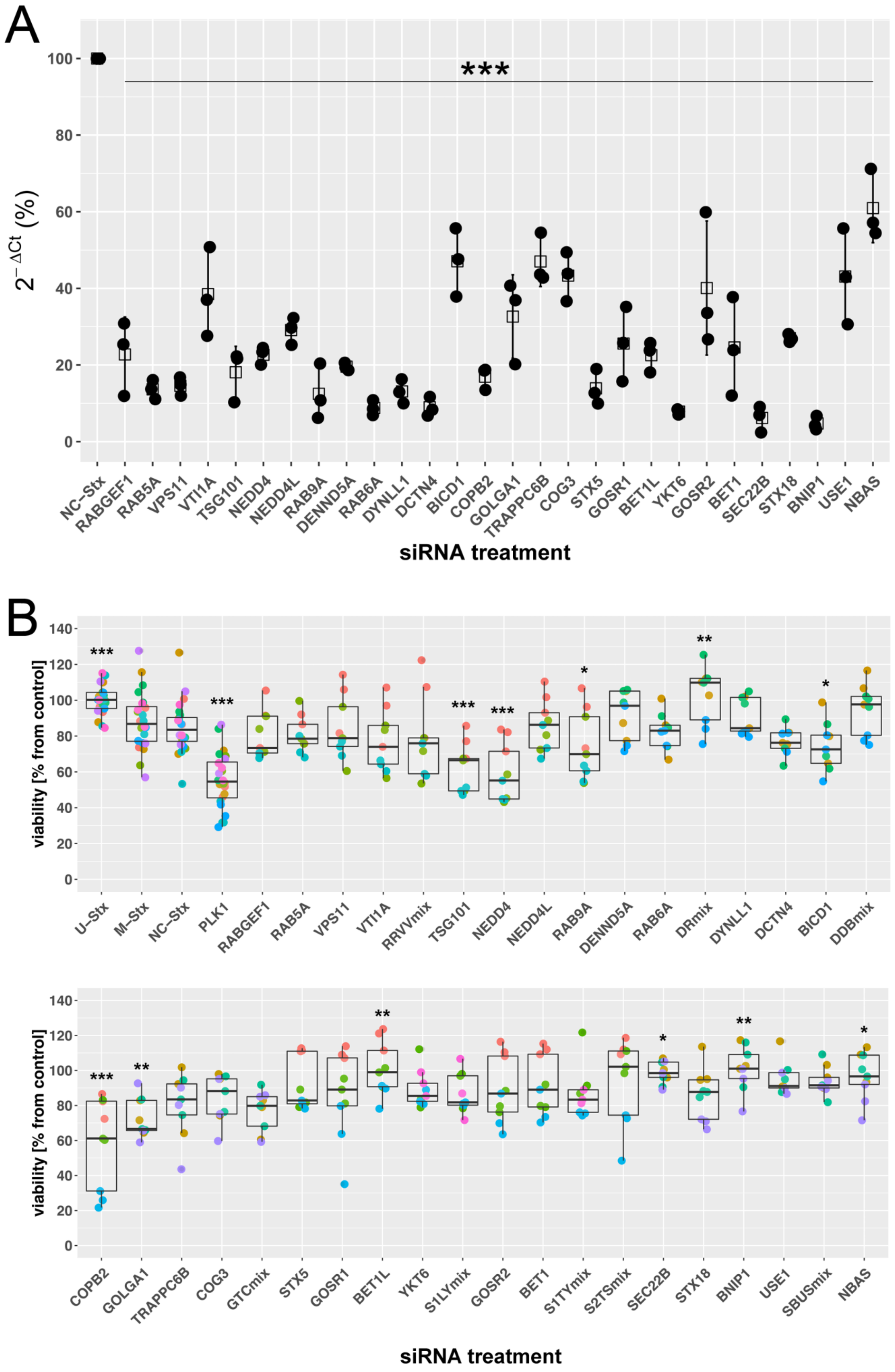
A
***
100
80
60
40
20
0
$2^{-\Delta Ct}$ (%)
NC-Stx
RABGEF1
RAB5A
VPS11
VTI1A
TSG101
NEDD4
NEDD4L
RAB9A
DENND5A
RAB6A
DYNLL1
DCTN4
BICD1
COPB2
GOLGA1
TRAPPC6B
COG3
STX5
GOSR1
BET1L
YKT6
GOSR2
BET1
SEC22B
STX18
BNIP1
USE1
NBAS
siRNA treatment
B
viability [% from control]
U-Stx
M-Stx
NC-Stx
PLK1
RABGEF1
RAB5A
VPS11
VTI1A
RRVVmix
TSG101
NEDD4
NEDD4L
RAB9A
DENND5A
RAB6A
DRmix
DYNLL1
DCTN4
BICD1
DDBmix
COPB2
GOLGA1
TRAPPC6B
COG3
GTCmix
STX5
GOSR1
BET1L
YKT6
S1LYmix
GOSR2
BET1
S1TYmix
S2TSmix
SEC22B
STX18
BNIP1
USE1
SBUSmix
NBAS
siRNA treatment

Figure S4. Effect of siRNA knockdowns on the viability of ACHN cells. (A) Assessment of siRNA knockdowns in ACHN cells by RT-PCR. ACHN cells were reverse transfected with a scrambled NC or the indicated siRNA(s). Extracted RNA was used in one-step RT-PCR targeting the respective host factor. Depicted are means with standard deviation for three biological replicates ($n = 3$) each performed in triplicate. A one-way ANOVA showed that the effect of siRNA type was significant ($F = 26.48$, $p < 2 \times 10^{-16}$). Multiple comparisons using *t*-tests with adjusted *p*-values (Holm method) revealed that all siRNA knockdowns were significantly different compared to NC (***, $p < 0.001$). For details, refer to Table S6. (B) ACHN cells were not transfected (U-Stx), mock-transfected (M-Stx), or reverse transfected with a scrambled NC (NC-Stx) or the indicated siRNA(s), respectively, all without Stx2a exposure. PLK1-targeted siRNAs served as a cell death control. Cell viability values are depicted as the percentage of untreated cells, which were set to 100% viability. Depicted are means with standard deviation for three biological replicates ($n = 3$) each performed in triplicate. Statistical analysis was performed using mixed effects models, and NC (NC-Stx) was used as the reference level. Significance: *, $p < 0.05$; **, $p < 0.01$; ***, $p < 0.001$.

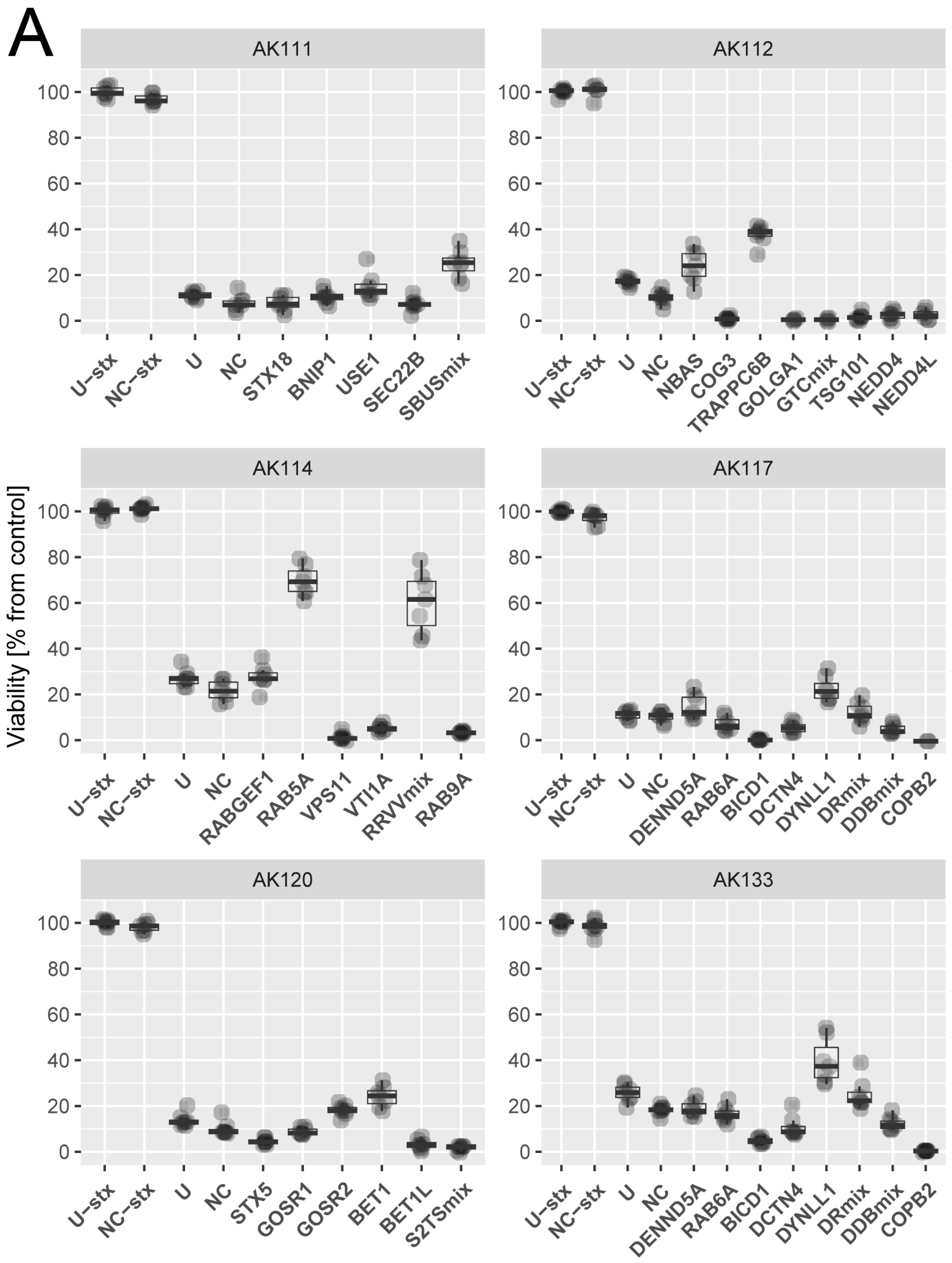
A
AK111
U−stx
NC−stx
U
NC
STX18
BNIP1
USE1
SEC22B
SBUSmix
AK112
U−stx
NC−stx
U
NC
NBAS
COG3
TRAPPC6B
GOLGA1
GTCmix
TSG101
NEDD4
NEDD4L
AK114
U−stx
NC−stx
U
NC
RABGEF1
RAB5A
VPS11
VTI1A
RRVVmix
RAB9A
AK117
U−stx
NC−stx
U
NC
DENND5A
RAB6A
BICD1
DCTN4
DYNLL1
DRmix
DDBmix
COPB2
AK120
U−stx
NC−stx
U
NC
STX5
GOSR1
GOSR2
BET1
BET1L
S2TSmix
AK133
U−stx
NC−stx
U
NC
DENND5A
RAB6A
BICD1
DCTN4
DYNLL1
DRmix
DDBmix
COPB2
Viability [% from control]
0
20
40
60
80
100

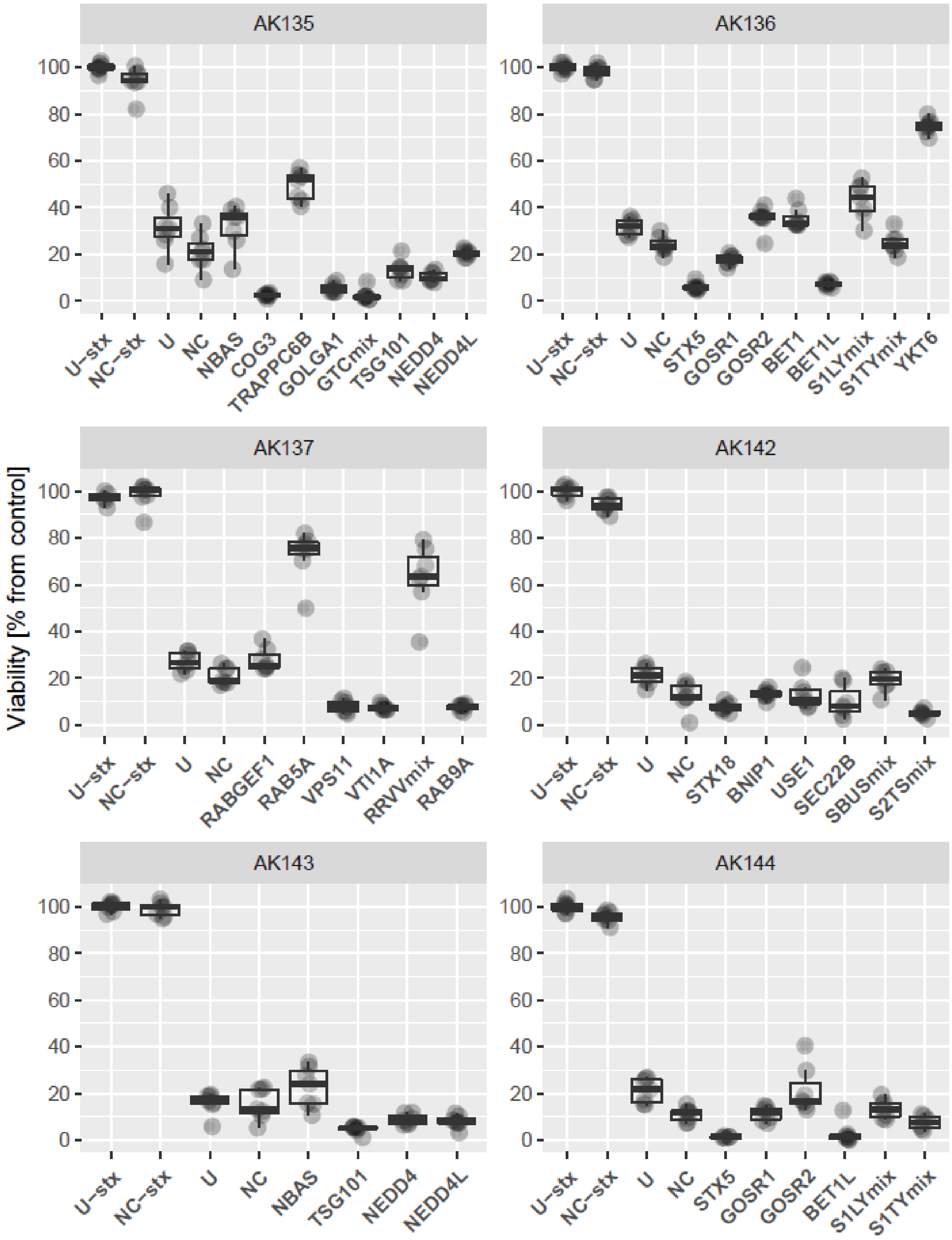
AK135
AK136
AK137
AK142
AK143
AK144
Viability [% from control]
0
20
40
60
80
100
U-stx
NC-stx
U
NC
NBAS
COG3
TRAPPC6B
GOLGA1
GTCmix
TSG101
NEDD4
NEDD4L
STX5
GOSR1
GOSR2
BET1
BET1L
S1LYmix
S1TYmix
YKT6
RABGEF1
RAB5A
VPS11
VTI1A
RRVVmix
RAB9A
STX18
BNIP1
USE1
SEC22B
SBUSmix
S2TSmix

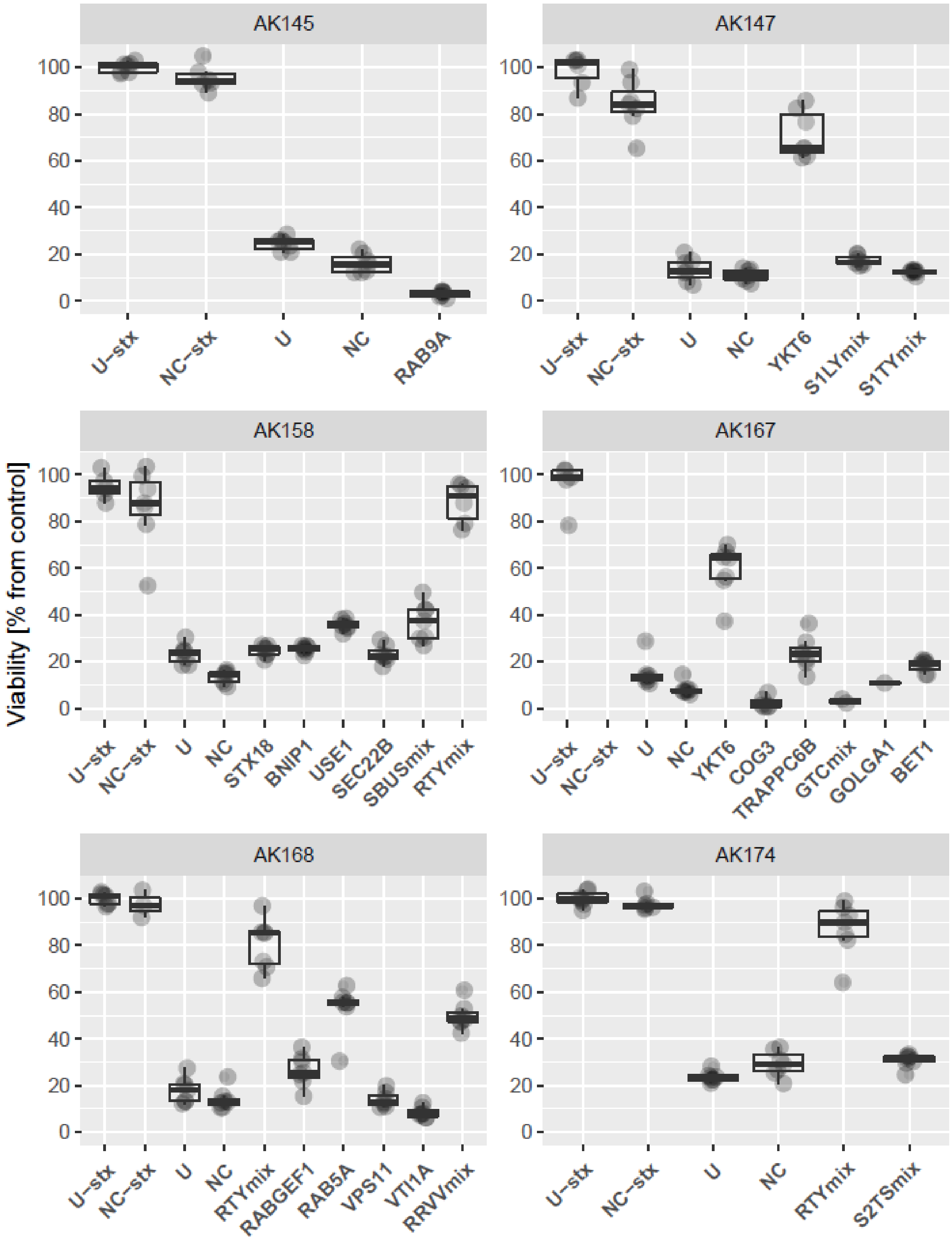
AK145
AK147
AK158
AK167
AK168
AK174
Viability [% from control]
0
20
40
60
80
100
U-stx
NC-stx
U
NC
RAB9A
YKT6
S1LYmix
S1TYmix
STX18
BNIP1
USE1
SEC22B
SBUSmix
RTYmix
COG3
TRAPPC6B
GTCmix
GOLGA1
BET1
RABGEF1
RAB5A
VPS11
VTI1A
RRVVmix
S2TSmix

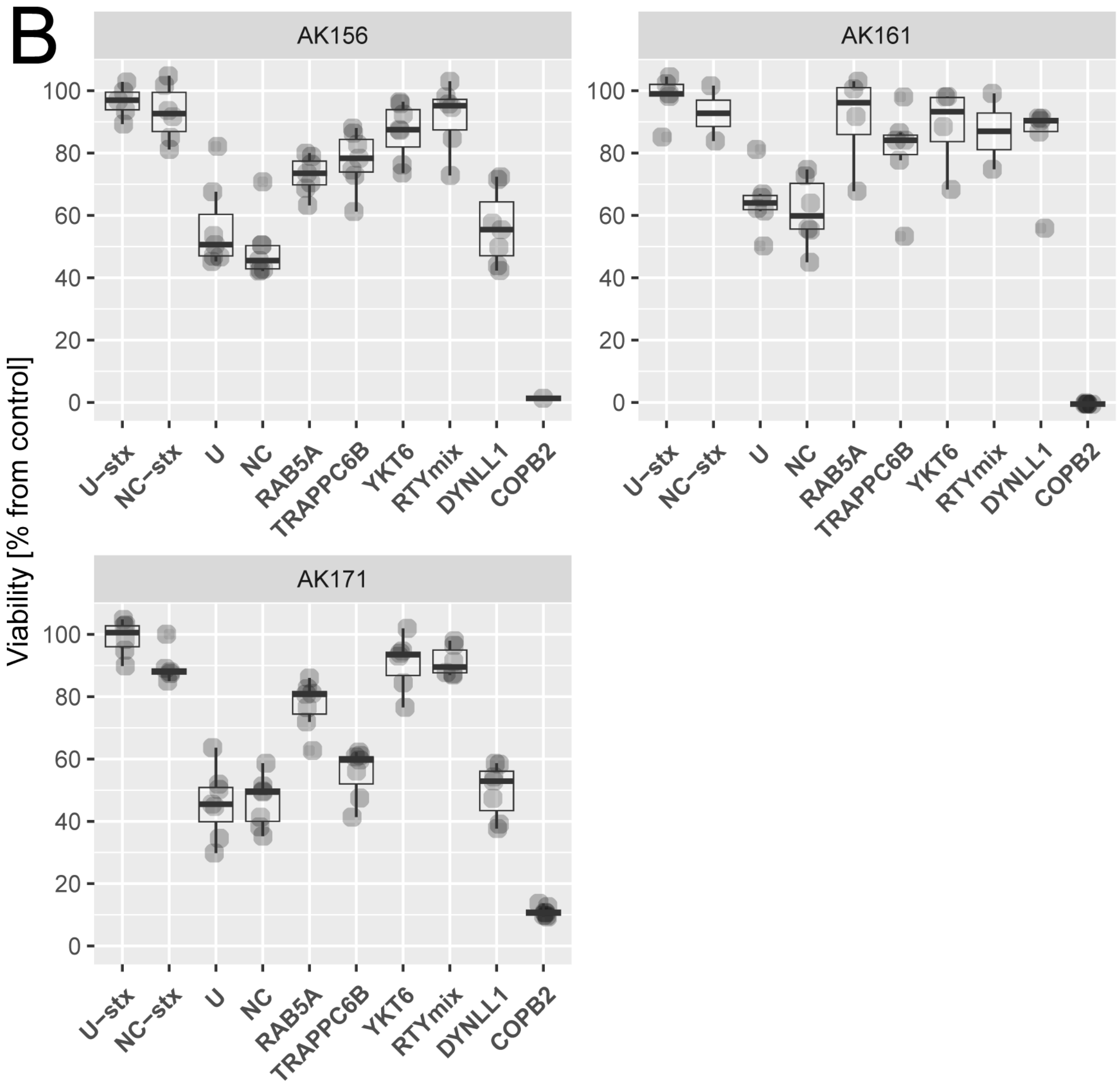

Figure S5. Survival of ACHN cells upon application of siRNA prior to Stx2a exposure including controls. ACHN cells were not transfected (U) or reverse transfected with a scrambled NC or the indicated siRNA(s). Then, cells were incubated without (-stx) or with Stx2a for 72 h (A) or 48 h (B). Cell viability values are depicted as the percentage of untreated cells, which were set to 100% viability. Depicted are three biological replicates (n = 3) each performed in septuplicate separated by their respective experimental groups denominated by laboratory-internal AK# designations.

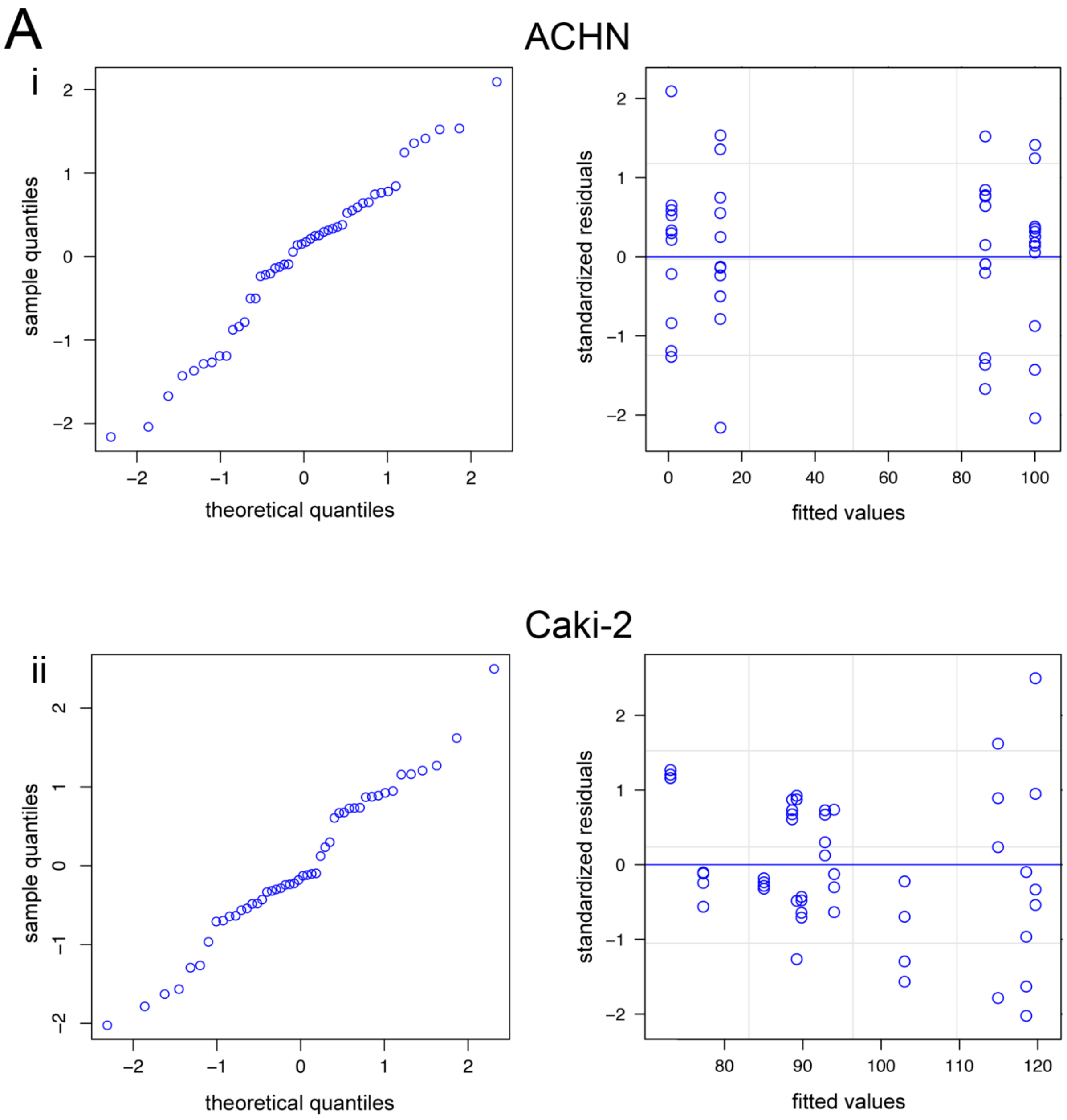
A
ACHN
i
sample quantiles
theoretical quantiles
standardized residuals
fitted values
Caki-2
ii
sample quantiles
theoretical quantiles
standardized residuals
fitted values

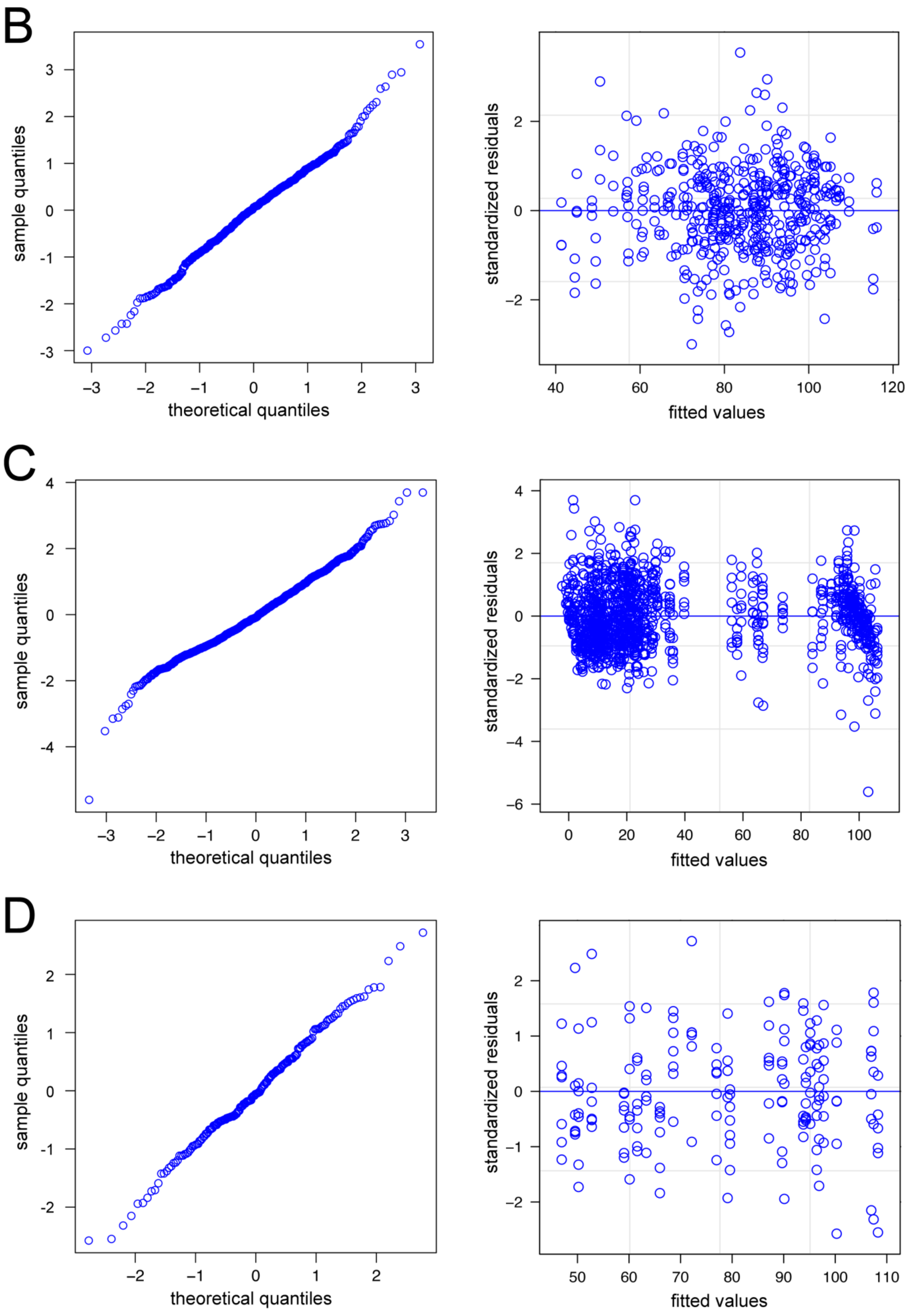
B
sample quantiles
theoretical quantiles
standardized residuals
fitted values
C
sample quantiles
theoretical quantiles
standardized residuals
fitted values
D
sample quantiles
theoretical quantiles
standardized residuals
fitted values

Figure S6. Mixed effects models validation for cytotoxicity tests that fit the observed viability. (A) Corresponding to data from **Error! Reference source not found.** in relation to the concentration of Stx2a for ACHN (i) and Caki-2 (ii) cells. (B) Corresponding to data from Figure S4B in relation to individual or mixed siRNA treatment. (C) Corresponding to data from **Error! Reference source not found.** in relation to siRNA treatment and Stx2a exposure for 72 h. (D) Corresponding to data from **Error! Reference source not found.** in relation to siRNA treatment and Stx2a exposure for 48 h. Left panels: quantile-quantile plots of the normalised residuals. The residuals are forming a nearly straight line, suggesting that they are normally distributed and the models are valid. Right panels: standardised residuals of the mixed effects models plotted against fitted values. There is no obvious pattern in points, suggesting that the difference in variance between treatment groups was taken into account and the models are valid.

Table S1. Summary of RNAseq data of Stx2a-treated ACHN and Caki-2 cells[a]

Sample[b]	PF Clusters[c]	Yield (Mbases)	% PF Clusters	% ≥ Q30 Bases	Mean Quality Score
ACHN Stx2a 4 h (1)	54,391,322	1,958	100	98.15	39.59
ACHN Stx2a 4 h (2)	50,820,262	1,830	100	98.11	39.57
ACHN Stx2a 4 h (3)	63,032,897	2,269	100	98.14	39.58
ACHN Stx2a 8 h (1)	61,938,281	2,230	100	98.23	39.6
ACHN Stx2a 8 h (2)	69,612,620	2,506	100	98.28	39.61
ACHN Stx2a 8 h (3)	58,457,119	2,104	100	98.28	39.61
ACHN control (1)	59,261,486	2,133	100	98.05	39.55
ACHN control (2)	42,372,772	1,525	100	98.13	39.58
ACHN control (3)	64,501,516	2,322	100	98.05	39.55
Caki-2 Stx2a 4 h (1)	53,994,370	1,944	100	98.34	39.63
Caki-2 Stx2a 4 h (2)	38,173,254	1,374	100	98.44	39.66
Caki-2 Stx2a 4 h (3)	75,556,555	2,720	100	98.34	39.63
Caki-2 Stx2a 8 h (1)	46,897,885	1,688	100	98.39	39.64
Caki-2 Stx2a 8 h (2)	72,755,615	2,619	100	98.34	39.63
Caki-2 Stx2a 8 h (3)	47,095,848	1,695	100	98.40	39.65
Caki-2 control (1)	50,498,374	1,818	100	98.28	39.61
Caki-2 control (2)	64,676,478	2,328	100	98.31	39.62
Caki-2 control (3)	43,332,454	1,560	100	98.33	39.63

[a]data were obtained by 36 bp single-end sequencing using an Illumina HiSeq3000
[b]numbers in parentheses indicate biological replicates of the experimental conditions. i.e. 4 h or 8 h exposure and untreated control
[c]clusters-passing filter

Table S2. References corresponding to selected host factors addressed in this study.

gene symbol	Reference
genes with higher expression in ACHN cells and a known role in Stx trafficking	
ATP6V0A1, ATP6V0D1, and APT6V1G1	[1-3]
BICD1	[4]
BNIP1	[5]
COG3	[5,6]
DYNLL1	[7]
GOLGA1	[8]
GOSR1	[5,9,10]
STX5	[9,10]
TBC1D17	[11]
VAMP2	[12]
VPS11	[13,14]
genes with higher expression in Caki-2 cells and a known role in Stx trafficking	
ARL1	[8,15]
BICD2	[4]
RAB11A	[16,17]
genes with higher expression in ACHN cells and an unknown role in Stx trafficking	
DCTN4	[18]
DENND5A	[19,20]
NEDD4	[21]
NEDD4L	[21]
RAB5A	[22,23]
RAB9A	[24]
RABGEF1	[25]
TRAPPC6B	[26]
TSG101	[21]
USE1	[27]
other genes analysed with a known role in Stx trafficking	
BET1L	[10]
NBAS	[5]
RAB6A	[28-30]
VTI1A	[5]
YKT6	[10]
other genes analysed with an unknown role in Stx trafficking	
BET1	[31,32]
COPB2	[28,33,34]
GOSR2	[31]
SEC22B	[27,31]
STX18	[35]

Table S3. UniProt-extracted data on 143 selected endosome-, Golgi- and ER-localised genes with higher expression in ACHN versus Caki-2 cells

See separate excel file. Corresponding to **Error! Reference source not found.**.

Table S4. Individual siRNAs used for RNAi and oligonucleotides used for RT-PCR

siRNAs		
**gene symbol**	**siRNA name**	**catalogue no.**
---	AllStars	SI03650318
BET1	Hs_BET1_11	SI04994542
BET1L	Hs_BET1L_9	SI04289929
BICD1	Hs_BICD1_5<br>Hs_BICD1_6<br>Hs_BICD1_7<br>Hs_BICD1_8<br>Hs_BICD1_9<br>Hs_BICD1_10	SI04250526<br>SI04256756<br>SI04364395<br>SI04367587<br>SI05006883<br>SI05006890
BNIP1	Hs_BNIP1_6	SI02777033
COG3	Hs_COG3_8<br>Hs_COG3_9	SI05099241<br>SI05099248
COPB2	Hs_COPB2_7<br>Hs_COPB2_9	SI02664816<br>SI05108740
DCTN4	Hs_DCTN4_1<br>Hs_DCTN4_2<br>Hs_DCTN4_4<br>Hs_DCTN4_5	SI00360437<br>SI00360444<br>SI00360458<br>SI04201757
DENND5A	Hs_RAB6IP1_5<br>Hs_RAB6IP1_6<br>Hs_RAB6IP1_7<br>Hs_RAB6IP1_8	SI03170573<br>SI03190180<br>SI04156551<br>SI04322073
DYNLL1	Hs_DYNLL1_3<br>Hs_DYNLL1_4<br>Hs_DYNLL1_5<br>Hs_DYNLL1_6	SI03167052<br>SI04238780<br>SI04271344<br>SI04323921
GEM	Hs_GEM_5	SI00162036
GOLGA1	Hs_GOLGA1_8<br>Hs_GOLGA1_9	SI04349786<br>SI04355659
GOSR1	Hs_GOSR1_7	SI03649072
GOSR2	Hs_GOSR2_16	SI05048743
NEDD4	Hs_NEDD4_8	SI03648925
NEDD4L	Hs_NEDD4L_10	SI05150271
NBAS	Hs_NAG_9	SI05041022
PLK1	Hs_PLK1_4<br>Hs_PLK1_6<br>Hs_PLK1_7<br>Hs_PLK1_11	SI00071638<br>SI02223837<br>SI02223844<br>SI04376365
RAB5A	Hs_RAB5A_5	SI00301588
RAB6A	Hs_RAB6A_6<br>Hs_RAB6A_7<br>Hs_RAB6A_8<br>Hs_RAB6A11	SI02655044<br>SI02654120<br>SI02655044<br>SI04437006
RAB9A	Hs_RAB9A_5	SI02663234
RABGEF1	Hs_RABGEF1_7<br>Hs_RABGEF1_8	SI04195877<br>SI04273479

SEC22B	Hs_SEC22B_5	SI04242511
STC1	Hs_STC1_4	SI00048496
STC2	Hs_STC2_5	SI02631706
STX5	Hs_STX5_1	SI03052966
STX18	Hs_STX18_10	SI05088524
TRAPPC6B	Hs_TRAPPC6B_7<br>Hs_TRAPPC6B_8	SI04158469<br>SI04349233
TSG101	Hs_TSG101_7	SI02664522
USE1	Hs_MDS032_6	SI04363674
VPS11	Hs_VPS11_6	SI02778167
VTI1A	Hs_VTI1A_11	SI04426443
YKT6	Hs_YKT6_6	SI04271085
Oligonucleotides		
**Targeted gene**	**Designation**	**Sequence 5'-3'**
BET1	Hs-BET1-qPCR-For<br>Hs-BET1-qPCR-Rev	AGTACCTCCTGGCAACTATGG<br>TCTTCACAGGCACTATACCCAC
BET1L	Hs-BET1L-qPCR-For<br>Hs-BET1L-qPCR-Rev	CTCGGATTTCACAAGCATGACC<br>GAAGGCCACAATTAGACCCAC
BICD1	Hs-BICD1-qPCR-For<br>Hs-BICD1-qPCR-Rev	TCCATCCACCGGAAGGTTG<br>GGCTCTGTTTCAGCTCGTTC
BNIP1	Hs-BNIP1-qPCR-For2<br>Hs-BNIP1-qPCR-Rev2	CCCGGCGATTGTATTTTGTGAT<br>TCAGGACAACCTAACAGATGCT
COG3	Hs-COG3-qPCR-For<br>Hs-COG3-qPCR-Rev	CAGGAACCCTACATGAAGCCT<br>GGCCAGCATAGGTATAAATCCGT
COPB2	Hs-COPB2-qPCR-For<br>Hs-COPB2-qPCR-Rev	CTTCCTGTTCGAGCTGCAAAG<br>CACTCTAATCTGCATGTCATCCG
DCTN4	Hs-DCTN4-qPCR-For<br>Hs-DCTN4-qPCR-Rev	CACACCCTCTCTACTCGGG<br>ACATGCCAGGTAATAGGCTTTC
DENND5A	Hs-DENND5A-qPCR-For<br>Hs-DENND5A-qPCR-Rev	AGGATGGCTCTCGGACATTTG<br>GGGGAGCATGTAGGACATCAT
DYNLL1	Hs-DYNLL1-qPCR-For<br>Hs-DYNLL1-qPCR-Rev	AGAGATGCAACAGGACTCGGT<br>CCAGGTGGGATTGTACTTCTTG
GAPDH	Hs-GAPDH-qPCR-For2<br>Hs-GAPDH-qPCR-Rev2	TGCACCACCAACTGCTTAGC<br>GGCATGGACTGTGGTCATGAG
GEM	Hs-GEM-qPCR-For<br>Hs-GEM-qPCR-Rev	GCAACCGCCATTCTGCTAC<br>CTCCCCTATGAGCACCACTC
GOLGA1	Hs-GOLGA1-qPCR-For<br>Hs-GOLGA1-qPCR-Rev	GGTCTGTGAGCAAGGAATCAG<br>GTTTCGGACCTGTTCAGCATAG
GOSR1	Hs-GOSR1-qPCR-For<br>Hs-GOSR1-qPCR-Rev	GCAAGGCTTACAGGGGTAAAT<br>CAGGGCTGCATTCAAGGAGG
GOSR2	Hs-GOSR2-qPCR-For<br>Hs-GOSR2-qPCR-Rev	CTTCGGGTTGACCAGTTAAAGT<br>AAGGTTCGAGACAGAAGCTCT
NBAS	Hs-NBAS-qPCR-For<br>Hs-NBAS-qPCR-Rev	CTGTTCAAGATCAGTGTGTGGAA<br>ACTCCATGCTACCCGTCTCC
NEDD4	Hs-NEDD4-qPCR-For2<br>Hs-NEDD4-qPCR-Rev2	TCAGGACAACCTAACAGATGCT<br>TTCTGCAAGATGAGTTGGAACAT
NEDD4L	Hs-NEDD4L-qPCR-For<br>Hs-NEDD4L-qPCR-Rev	GACATGGAGCATGGATGGGAA<br>GTTCGGCCTAAATTGTCCACT
RAB5A	Hs-RAB5A-qPCR-For<br>Hs-RAB5A-qPCR-Rev	AGACCCAACGGGCCAAATAC<br>GCCCCAATGGTACTCTCTTGAA

RAB6A	Hs-RAB6A-qPCR-For<br>Hs-RAB6A-qPCR-Rev	CTTGGAGGATCGAACAGTACGA<br>AGCTAGGAATCAAGCTCCTGAA
RAB9A	Hs-RAB9A-qPCR-For<br>Hs-RAB9A-qPCR-Rev	AGGGACAACGGCGACTATC<br>TCTGACCTATCCTCGGTAGCA
RABGEF1	Hs-RABGEF1-qPCR-For<br>Hs-RABGEF1-qPCR-Rev	ATGTGGATCAATCGGATCTCCT<br>GCTTTGTGGTACTCTTCCCTCC
SEC22B	Hs-SEC22B-qPCR-For<br>Hs-SEC22B-qPCR-Rev	AGAAGTTGGCTTTTGCCTACC<br>CACGACTGTCAATGTAGAGCTT
STC1	Hs-STC1-qPCR-For<br>Hs-STC1-qPCR-Rev	AGGTGCAGGAAGAGTGCTACA<br>GACGACCTCAGTGATGGCTT
STC2	Hs-STC2-qPCR-For2<br>Hs-STC2-qPCR-Rev2	ACAGGTTCGGCTGCATAAGC<br>GAGGTCCACGTAGGGTTCG
STX5	Hs-STX5-qPCR-For<br>Hs-STX5-qPCR-Rev	TTTCCAGCGTTGTGCAGAAAA<br>AAAGCGCAAGTCCCTCTTTGA
STX18	Hs-STX18-qPCR-For<br>Hs-STX18-qPCR-Rev	AACGAGACCAGATAGACCAGG<br>CCTTGTGAGCTTCTGTTCGTAG
TRAPPC6B	Hs-TRAPPC6B-qPCR-For<br>Hs-TRAPPC6B-qPCR-Rev	GATGAGGCGTTGTTTTTGCTT<br>CCCACTCGAAACCCCATGTT
TSG101	Hs-TSG101-qPCR-For<br>Hs-TSG101-qPCR-Rev	GAGAGCCAGCTCAAGAAAATGG<br>TGAGGTTCATTAGTTCCCTGGA
USE1	Hs-USE1-qPCR-For<br>Hs-USE1-qPCR-Rev	GAGATGCGGAGTGAGCTACTA<br>CCAGGGTATTGGTCTTGAGGC
VPS11	Hs-VPS11-qPCR-For2<br>Hs-VPS11-qPCR-Rev2	CAATCCACTCTGCACTCGAAT<br>CGGGTGATGTCTCCTTTGTTCA
VTI1A	Hs-VTI1A-qPCR-For<br>Hs-VTI1A-qPCR-Rev	CCAGCAAGATTGCGAGGGT<br>GCAGTTCTTTCGCTTCTTCAAG
YKT6	Hs-YKT6-qPCR-For<br>Hs-YKT6-qPCR-Rev	CAGCGTCCTCTACAAAGGCG<br>ACAATCAGTTGACTCGTGAAGG

Table S5. Model coefficients summary of the regression analysis

Analysis of changes in ACHN and Caki-2 cell viability in response to the Stx2a concentration corresponding to *Error! Reference source not found.*				
Condition[a]	***Estimate[b]***	***Std. Error***	***t-value***	***p-value[c]***
**ACHN control (intercept)**	**100.00**	**1.99**	**50.27**	**0.0000 (***)**
**AHCN Stx2a 0.5 pg/mL**	**-13.57**	**3.61**	**-3.76**	**0.0005 (***)**
**AHCN Stx2a 0.5 ng/mL**	**-85.87**	**2.54**	**-33.82**	**0.0000 (***)**
**AHCN Stx2a 0.5 µg/mL**	**-99.19**	**2.00**	**-49.65**	**0.0000 (***)**
**Caki-2 control (intercept)**	**100.00**	**10.44**	**9.58**	**0.0000 (***)**
Caki-2 Stx2a 0.5 pg/mL	1.18	4.75	0.25	0.8055 (n.s.)
Caki-2 Stx2a 0.5 ng/mL	-3.61	4.90	-0.73	0.4654 (n.s.)
**Caki-2 Stx2a 0.5 µg/mL**	**-15.54**	**5.72**	**-2.72**	**0.0095 (**)**
Analysis of the siRNA treatment effect on the ACHN cell viability corresponding to Figure S4B				
Condition[a]	***Estimate[d]***	***Std. Error***	***t-value***	***p-value[c]***
**NC-Stx (intercept)**	**85.00**	**4.05**	**20.97**	**0.0000 (***)**
**U-Stx**	**15.00**	**3.62**	**4.15**	**0.0000 (***)**
M-Stx	3.50	3.58	0.98	0.3284 (n.s.)
**PLK1**	**-29.75**	**3.58**	**-8.32**	**0.0000 (***)**
RABGEF1	-4.51	5.02	-0.90	0.3694 (n.s.)
RAB5A	-4.08	5.02	-0.81	0.4169 (n.s.)
VPS11	-0.70	5.02	-0.14	0.8892 (n.s.)
VTI1A	-7.66	5.02	-1.53	0.1272 (n.s.)
RRVVmix	-6.51	5.02	-1.30	0.1949 (n.s.)
**TSG101**	**-22.53**	**5.02**	**-4.50**	**0.0000 (***)**
**NEDD4**	**-26.14**	**5.02**	**-5.21**	**0.0000 (***)**
NEDD4L	1.084	5.02	0.22	0.8290 (n.s.)
**RAB9A**	**-10.51**	**5.02**	**-2.10**	**0.0367 (*)**
DENND5A	5.37	5.08	1.06	0.2907 (n.s.)
RAB6A	-3.37	5.08	-0.66	0.5069 (n.s.)
**DRmix**	**16.31**	**5.08**	**3.21**	**0.0014 (**)**
DYNLL1	4.82	5.08	0.95	0.3428 (n.s.)
DCTN4	-8.94	5.08	-1.76	0.0789 (n.s.)
**BICD1**	**-11.76**	**5.08**	**-2.32**	**0.0210 (*)**
DDBmix	8.75	5.08	1.72	0.0855 (n.s.)
**COPB2**	**-26.60**	**5.02**	**-5.30**	**0.0000 (***)**
**GOLGA1**	**-13.58**	**5.08**	**-2.68**	**0.0078 (**)**
TRAPPC6B	-5.50	5.08	-1.08	0.2787 (n.s.)
COG3	-1.76	5.08	-0.34	0.7295 (n.s.)
GTCmix	-9.10	5.08	-1.79	0.0736 (n.s.)
STX5	6.99	5.02	1.39	0.1640 (n.s.)
GOSR1	1.14	5.02	0.23	0.8207 (n.s.)
**BET1L**	**15.81**	**5.02**	**3.15**	**0.0017 (*)**
YKT6	8.94	4.99	1.80	0.0738 (n.s.)
S1LYmix	6.46	4.99	1.30	0.1959 (n.s.)
GOSR2	3.95	5.02	0.79	0.4316 (n.s.)
BET1	6.26	5.02	1.25	0.2127 (n.s.)
S1TYmix	6.21	4.99	1.25	0.2134 (n.s.)
S2TSmix	9.25	5.02	1.84	0.0659 (n.s.)

**SEC22B**	**12.60**	**5.08**	**2.48**	**0.0134 (*)**
STX18	-0.10	5.08	-0.02	0.9842 (n.s.)
**BNIP1**	**15.00**	**5.08**	**2.95**	**0.0033 (**)**
USE1	9.18	5.08	1.81	0.0711 (n.s.)
SBUSmix	7.97	5.08	1.57	0.1172 (n.s.)
**NBAS**	**10.38**	**5.08**	**2.04**	**0.0415 (*)**
Analysis of the siRNA treatment and 72 h Stx2a exposure effect on the ACHN cell viability corresponding to *Error! Reference source not found.*				
Condition[e]	***Estimate[f]***	***Std. Error***	***t-value***	***p-value***
NC+Stx (intercept)	15.25	1.04	14.62	0.0000
U-Stx	84.75	0.71	119.96	0.0000
NC-Stx	82.26	0.94	87.21	0.0000
U+Stx	5.38	0.74	7.25	0.0000
RABGEF1	9.66	1.43	6.77	0.0000
**RAB5A**	**47.42**	**2.84**	**16.67**	**0.0000**
VPS11	-9.97	1.36	-7.35	0.0000
VTI1A	-10.70	0.84	-12.79	0.0000
**RRVVmix**	**40.08**	**2.84**	**14.11**	**0.0000**
TSG101	-8.39	1.14	-7.39	0.0000
NEDD4	-7.69	0.92	-8.34	0.0000
NEDD4L	-4.66	1.51	-3.09	0.0020
RAB9A	-12.27	0.77	-16.00	0.0000
DENND5A	2.46	1.50	1.64	0.1016
RAB6A	-2.63	1.41	-1.86	0.0628
DRmix	4.22	2.08	2.03	0.0423
**DYNLL1**	**16.46**	**2.85**	**5.78**	**0.0000**
DCTN4	-6.28	1.32	-4.77	0.0000
BICD1	-11.92	0.94	-12.72	0.0000
DDBmix	-5.81	1.19	-4.88	0.0000
COPB2	-14.26	1.09	-13.03	0.0000
**RTYmix**	**66.06**	**2.47**	**26.70**	**0.0000**
GOLGA1	-12.13	0.90	-13.47	0.0000
**TRAPPC6B**	**22.30**	**2.49**	**8.94**	**0.0000**
COG3	-12.72	0.87	-14.62	0.0000
GTCmix	-13.32	0.86	-15.47	0.0000
STX5	-11.92	0.94	-12.62	0.0000
GOSR1	-3.10	0.91	-3.40	0.0007
BET1L	-11.38	1.01	-11.28	0.0000
**YKT6**	**53.30**	**2.35**	**22.65**	**0.0000**
S1LYmix	9.51	2.52	3.77	0.0002
GOSR2	9.26	1.82	5.08	0.0000
BET1	10.07	1.42	7.10	0.0000
S1TYmix	-0.56	1.46	-0.38	0.7005
S2TSmix	-2.78	2.13	-1.30	0.1922
SEC22B	0.51	1.30	0.39	0.6986
STX18	0.27	1.10	0.24	0.8090
BNIP1	3.35	0.92	3.63	0.0003
USE1	8.17	1.69	4.82	0.0000
SBUSmix	14.04	1.74	8.05	0.0000

NBAS	11.15	1.99	5.60	0.0000
Analysis of the siRNA treatment and 48 h Stx2a exposure effect on the ACHN cell viability corresponding to *Error! Reference source not found.*				
**Condition**	**Estimate**[f]	**Std. Error**	***t*-value**	***p*-value**[c]
NC+Stx (intercept)	52.20	4.63	11.28	0.0000 (***)
U-Stx	48.20	2.94	16.38	0.0000 (***)
NC-Stx	46.87	3.21	14.61	0.0000 (***)
U+Stx	3.24	3.39	0.95	0.3412 (n.s.)
RAB5A	30.03	3.34	8.99	0.0000 (***)
TRAPPC6B	19.03	3.68	5.18	0.0000 (***)
YKT6	40.17	3.50	11.48	0.0000 (***)
RTYmix	47.34	3.81	12.44	0.0000 (***)
DYNLL1	12.06	4.53	2.66	0.0085 (**)

[a]significantly different conditions in bold
[b]values for the respective controls are set as 100% and all other values denote the changes in comparison to the respective control
[c]significance levels in parentheses: *, $p < 0.05$; **, $p < 0.01$; ***, $p < 0.001$; n.s., not significant
[d]values for NC-Stx are in relation to U-Stx which is set as 100% and all other values denote the changes in comparison to NC-Stx
[e]significantly different conditions with at least 15% difference to NC+Stx and 30% viability in bold
[f]values for NC+Stx are in relation to U-Stx which is set as 100% and all other values denote the changes in comparison to NC+Stx

Table S6. Pairwise comparisons summary of the scrambled NC vs. the indicated siRNAs using *t*-tests[a]

Comparison	*p*-value (adjusted)[b]
NC-Stx vs. RABGEF1	2.8e-16 (***)
NC-Stx vs. RAB5A	< 2e-16 (***)
NC-Stx vs. VPS11	< 2e-16 (***)
NC-Stx vs. VTI1A	3.0e-12 (***)
NC-Stx vs. TSG101	< 2e-16 (***)
NC-Stx vs. NEDD4	2.6e-16 (***)
NC-Stx vs. NEDD4L	1.1e-14 (***)
NC-Stx vs. RAB9A	< 2e-16 (***)
NC-Stx vs. DENND5A	< 2e-16 (***)
NC-Stx vs. RAB6A	< 2e-16 (***)
NC-Stx vs. DYNLL1	< 2e-16 (***)
NC-Stx vs. DCTN4	< 2e-16 (***)
NC-Stx vs. BICD1	6.6e-10 (***)
NC-Stx vs. COPB2	< 2e-16 (***)
NC-Stx vs. GOLGA1	8.4e-14 (***)
NC-Stx vs. TRAPPC6B	6.4e-10 (***)
NC-Stx vs. COG3	6.1e-11 (***)
NC-Stx vs. STX5	< 2e-16 (***)
NC-Stx vs. GOSR1	1.4e-15 (***)
NC-Stx vs. BET1L	2.5e-16 (***)
NC-Stx vs. YKT6	< 2e-16 (***)
NC-Stx vs. GOSR2	8.0e-12 (***)
NC-Stx vs. BET1	7.7e-16 (***)
NC-Stx vs. SEC22B	< 2e-16 (***)
NC-Stx vs. STX18	3.1e-15 (***)
NC-Stx vs. BNIP1	< 2e-16 (***)
NC-Stx vs. USE1	5.3e-11 (***)
NC-Stx vs. NBAS	5.2e-06 (***)

[a]corresponding to Figure S4; the *p*-values are adjusted for multiple testing using the Holm method
[b]significance levels in parentheses: ***, $p < 0.001$

References

1 Dyve Lingelem, A. B., Bergan, J. & Sandvig, K. Inhibitors of intravesicular acidification protect against Shiga toxin in a pH-independent manner. *Traffic* **13**, 443-454, doi:10.1111/j.1600-0854.2011.01319.x (2012).

2 Moreau, D. *et al.* Genome-wide RNAi screens identify genes required for Ricin and PE intoxications. *Dev Cell* **21**, 231-244, doi:10.1016/j.devcel.2011.06.014 (2011).

3 Schapiro, F. B., Lingwood, C., Furuya, W. & Grinstein, S. pH-independent retrograde targeting of glycolipids to the Golgi complex. *Am J Physiol* **274**, C319-332 (1998).

4 Matanis, T. *et al.* Bicaudal-D regulates COPI-independent Golgi-ER transport by recruiting the dynein-dynactin motor complex. *Nat Cell Biol* **4**, 986-992, doi:10.1038/ncb891 (2002).

5 Yamaji, T. *et al.* A CRISPR Screen Identifies LAPTM4A and TM9SF Proteins as Glycolipid-Regulating Factors. *iScience* **11**, 409-424, doi:10.1016/j.isci.2018.12.039 (2019).

6 Zolov, S. N. & Lupashin, V. V. Cog3p depletion blocks vesicle-mediated Golgi retrograde trafficking in HeLa cells. *J Cell Biol* **168**, 747-759, doi:10.1083/jcb.200412003 (2005).

7 Hehnly, H., Sheff, D. & Stamnes, M. Shiga toxin facilitates its retrograde transport by modifying microtubule dynamics. *Mol Biol Cell* **17**, 4379-4389, doi:10.1091/mbc.E06-04-0310 (2006).

8 Lu, L., Tai, G. & Hong, W. Autoantigen Golgin-97, an effector of Arl1 GTPase, participates in traffic from the endosome to the *trans*-Golgi network. *Mol Biol Cell* **15**, 4426-4443, doi:10.1091/mbc.E03-12-0872 (2004).

9 Amessou, M. *et al.* Syntaxin 16 and syntaxin 5 are required for efficient retrograde transport of several exogenous and endogenous cargo proteins. *J Cell Sci* **120**, 1457-1468, doi:10.1242/jcs.03436 (2007).

10 Tai, G. *et al.* Participation of the syntaxin 5/Ykt6/GS28/GS15 SNARE complex in transport from the early/recycling endosome to the *trans*-Golgi network. *Mol Biol Cell* **15**, 4011-4022, doi:10.1091/mbc.E03-12-0876 (2004).

11 Fuchs, E. *et al.* Specific Rab GTPase-activating proteins define the Shiga toxin and epidermal growth factor uptake pathways. *J Cell Biol* **177**, 1133-1143, doi:10.1083/jcb.200612068 (2007).

12 Renard, H. F., Garcia-Castillo, M. D., Chambon, V., Lamaze, C. & Johannes, L. Shiga toxin stimulates clathrin-independent endocytosis of the VAMP2, VAMP3 and VAMP8 SNARE proteins. *J Cell Sci* **128**, 2891-2902, doi:10.1242/jcs.171116 (2015).

13 Kvalvaag, A. S., Pust, S. & Sandvig, K. Vps11, a subunit of the tethering complexes HOPS and CORVET, is involved in regulation of glycolipid degradation and retrograde toxin transport. *Commun Integr Biol* **7**, e28129, doi:10.4161/cib.28129 (2014).

14 Kvalvaag, A. S. *et al.* The ERM proteins ezrin and moesin regulate retrograde Shiga toxin transport. *Traffic* **14**, 839-852, doi:10.1111/tra.12077 (2013).

15 Nishimoto-Morita, K. *et al.* Differential effects of depletion of ARL1 and ARFRP1 on membrane trafficking between the *trans*-Golgi network and endosomes. *J Biol Chem* **284**, 10583-10592, doi:10.1074/jbc.M900847200 (2009).

16 McKenzie, J. E. *et al.* Retromer Guides STxB and CD8-M6PR from Early to Recycling Endosomes, EHD1 Guides STxB from Recycling Endosome to Golgi. *Traffic* **13**, 1140-1159, doi:10.1111/j.1600-0854.2012.01374.x (2012).

17 Wilcke, M. *et al.* Rab11 regulates the compartmentalization of early endosomes required for efficient transport from early endosomes to the *trans*-Golgi network. *J Cell Biol* **151**, 1207-1220 (2000).

18 Schroer, T. A. Dynactin. *Annu Rev Cell Dev Biol* **20**, 759-779 (2004).

19 Fernandes, H. *et al.* Structural aspects of Rab6-effector complexes. *Biochem Soc Trans* **37**, 1037-1041, doi:10.1042/BST0371037 (2009).

20 Fukuda, M., Kobayashi, H., Ishibashi, K. & Ohbayashi, N. Genome-wide investigation of the Rab binding activity of RUN domains: development of a novel tool that specifically traps GTP-Rab35. *Cell Struct Funct* **36**, 155-170 (2011).

21 McCullough, J., Colf, L. A. & Sundquist, W. I. Membrane fission reactions of the mammalian ESCRT pathway. *Annu Rev Biochem* **82**, 663-692, doi:10.1146/annurev-biochem-072909-101058 (2013).

22 Huotari, J. & Helenius, A. Endosome maturation. *EMBO J* **30**, 3481-3500, doi:10.1038/emboj.2011.286 (2011).

23 Hutagalung, A. H. & Novick, P. J. Role of Rab GTPases in membrane traffic and cell physiology. *Physiol Rev* **91**, 119-149, doi:10.1152/physrev.00059.2009 (2011).

24 Lombardi, D. *et al.* Rab9 functions in transport between late endosomes and the *trans* Golgi network. *EMBO J* **12**, 677-682 (1993).

25 Horiuchi, H. *et al.* A novel Rab5 GDP/GTP exchange factor complexed to Rabaptin-5 links nucleotide exchange to effector recruitment and function. *Cell* **90**, 1149-1159 (1997).

26 Yu, S. & Liang, Y. A trapper keeper for TRAPP, its structures and functions. *Cell Mol Life Sci* **69**, 3933-3944, doi:10.1007/s00018-012-1024-3 (2012).

27 Jahn, R. & Scheller, R. H. SNAREs--engines for membrane fusion. *Nat Rev Mol Cell Biol* **7**, 631-643, doi:10.1038/nrm2002 (2006).

28 Del Nery, E. *et al.* Rab6A and Rab6A' GTPases play non-overlapping roles in membrane trafficking. *Traffic* **7**, 394-407, doi:10.1111/j.1600-0854.2006.00395.x (2006).

29 Mallard, F. *et al.* Early/recycling endosomes-to-TGN transport involves two SNARE complexes and a Rab6 isoform. *J Cell Biol* **156**, 653-664, doi:10.1083/jcb.200110081 (2002).

30 Miserey-Lenkei, S. *et al.* Rab and actomyosin-dependent fission of transport vesicles at the Golgi complex. *Nat Cell Biol* **12**, 645-654, doi:10.1038/ncb2067 (2010).

31 Xu, D., Joglekar, A. P., Williams, A. L. & Hay, J. C. Subunit structure of a mammalian ER/Golgi SNARE complex. *J Biol Chem* **275**, 39631-39639, doi:10.1074/jbc.M007684200 (2000).

32 Zhang, T. & Hong, W. Ykt6 forms a SNARE complex with syntaxin 5, GS28, and Bet1 and participates in a late stage in endoplasmic reticulum-Golgi transport. *J Biol Chem* **276**, 27480-27487, doi:10.1074/jbc.M102786200 (2001).

33 Girod, A. *et al.* Evidence for a COP-I-independent transport route from the Golgi complex to the endoplasmic reticulum. *Nat Cell Biol* **1**, 423-430, doi:10.1038/15658 (1999).

34 White, J. *et al.* Rab6 coordinates a novel Golgi to ER retrograde transport pathway in live cells. *J Cell Biol* **147**, 743-760 (1999).

35 Hatsuzawa, K. *et al.* Syntaxin 18, a SNAP receptor that functions in the endoplasmic reticulum, intermediate compartment, and *cis*-Golgi vesicle trafficking. *J Biol Chem* **275**, 13713-13720 (2000).
